# Microbiota regulate intestinal epithelial gene expression by suppressing the transcription factor Hepatocyte nuclear factor 4 alpha

**DOI:** 10.1101/gr.220111.116

**Published:** 2017-07

**Authors:** James M. Davison, Colin R. Lickwar, Lingyun Song, Ghislain Breton, Gregory E. Crawford, John F. Rawls

**Affiliations:** 1 Department of Molecular Genetics and Microbiology, Center for the Genomics of Microbial Systems, Duke University, Durham, North Carolina 27710, USA;; 2 Department of Cell Biology and Physiology, University of North Carolina, Chapel Hill, North Carolina 27599, USA;; 3 Department of Pediatrics, Division of Medical Genetics, Center for Genomic and Computational Biology, Duke University, Durham, North Carolina 27708, USA;; 4 Department of Integrative Biology and Pharmacology, McGovern Medical School, Houston, Texas 77030, USA

## Abstract

Microbiota influence diverse aspects of intestinal physiology and disease in part by controlling tissue-specific transcription of host genes. However, host genomic mechanisms mediating microbial control of intestinal gene expression are poorly understood. Hepatocyte nuclear factor 4 (HNF4) is the most ancient family of nuclear receptor transcription factors with important roles in human metabolic and inflammatory bowel diseases, but a role in host response to microbes is unknown. Using an unbiased screening strategy, we found that zebrafish Hnf4a specifically binds and activates a microbiota-suppressed intestinal epithelial transcriptional enhancer. Genetic analysis revealed that zebrafish *hnf4a* activates nearly half of the genes that are suppressed by microbiota, suggesting microbiota negatively regulate Hnf4a. In support, analysis of genomic architecture in mouse intestinal epithelial cells disclosed that microbiota colonization leads to activation or inactivation of hundreds of enhancers along with drastic genome-wide reduction of HNF4A and HNF4G occupancy. Interspecies meta-analysis suggested interactions between HNF4A and microbiota promote gene expression patterns associated with human inflammatory bowel diseases. These results indicate a critical and conserved role for HNF4A in maintaining intestinal homeostasis in response to microbiota.

All animals face the fundamental challenge of building and maintaining diverse tissues while remaining sensitive and responsive to their environment. This is most salient in the intestinal epithelium which performs important roles in nutrient absorption and barrier function while being constantly exposed to complex microbial communities (microbiota) and nutrients within the intestinal lumen. The presence and composition of microbiota in the intestinal lumen influence diverse aspects of intestinal development and physiology including dietary nutrient metabolism and absorption, intestinal epithelial renewal, and edification of the host immune system. Abnormal host-microbiota interactions are strongly implicated in the pathogenesis of inflammatory bowel diseases (IBD), including Crohn's disease (CD) and ulcerative colitis (UC) (Sartor and Wu 2016). Studies in mouse and zebrafish models of IBD have established that impaired intestinal epithelial cell (IEC) responses to microbiota are a key aspect of disease progression (Bates et al. 2007; Kamada et al. 2013; Marjoram et al. 2015). Improved understanding of the molecular mechanisms by which microbiota evoke host responses in the intestinal epithelium can be expected to lead to new strategies for preventing or treating IBD and other microbiota-associated diseases.

The ability of IECs to maintain their physiologic functions and respond appropriately to microbial stimuli is facilitated through regulation of gene transcription. Genome-wide comparison of transcript levels in intestinal tissue or isolated IECs from mice reared in the absence of microbes (germ-free or GF) to those colonized with a microbiota (conventionalized or CV) have revealed hundreds of genes that have significantly increased or decreased mRNA levels following microbiota colonization (Camp et al. 2014). Interestingly, many mouse genes that are transcriptionally regulated by microbiota have zebrafish homologs that are similarly responsive, suggesting the existence of evolutionarily conserved regulatory mechanisms (Rawls et al. 2004). For example, the protein hormone Angiopoetin-like 4 (ANGPTL4, also called FIAF) is encoded by a single ortholog in the mouse and zebrafish genomes, and microbiota colonization results in significant reductions in transcript levels in the intestinal epithelium of both host species (Bäckhed et al. 2004; Camp et al. 2012). Whereas these impacts of microbiota on host IEC transcriptomes and their downstream consequences have been extensively documented, the upstream transcriptional regulatory mechanisms remain poorly understood.

Specification and tuning of gene transcription proceeds, in part, through interactions between transcription factors (TFs) and their sequence-specific binding to *cis*-regulatory DNA. *Cis*-regulatory regions (CRRs) harbor binding sites for multiple activating or repressing TFs and are generally associated with nucleosome depletion and specific post-translational modifications of histone proteins within adjacent nucleosomes when acting as poised (H3K4me1) or active (H3K27ac) enhancers (Creyghton et al. 2010). Antibiotic administration can impact transcript levels and histone modifications in IECs (Thaiss et al. 2016); however, it's unclear if these changes are indirect effects caused by alterations to microbiota composition, direct effects of the antibiotic on host cells, or the effects of remaining antibiotic-resistant microbiota (Morgun et al. 2015). Previous studies have shown that histone deacetylase 3 is required in IECs to maintain intestinal homeostasis in the presence of microbiota (Alenghat et al. 2013) and that overall histone acetylation and methylation in the intestine is altered by microbiota colonization (Krautkramer et al. 2016). However, the direct and specific effects of the microbiota on host CRRs and subsequent transcriptional responses in IECs remain unknown.

Our previous studies predicted key roles for one or more nuclear receptor (NR) TFs in microbial down-regulation of IEC gene expression (Camp et al. 2014), but the specific TF(s) were not identified. Nuclear receptors are ideal candidate TFs for integrating microbe-derived signals since, for many, their transcriptional activity can be positively or negatively regulated by the binding of metabolic or hormonal ligands (Evans and Mangelsdorf 2014). However, the roles of nuclear receptors in host responses remain poorly understood, and no previous study has defined the impact of microbiota on nuclear receptor DNA binding. Nuclear receptors are a metazoan innovation. The earliest animals encoded a single nuclear receptor orthologous to Hepatocyte nuclear factor 4 (HNF4; nuclear receptor subfamily NR2A) (Bridgham et al. 2010). Despite subsequent duplication and diversification, distinct HNF4 TFs remain encoded in extant animals including mammals (HNF4A, HNF4G) and fishes (Hnf4a, Hnf4b, Hnf4g) (Supplemental Fig. S1G). HNF4A serves particularly important roles in IECs, where it binds CRRs and activates expression of genes involved in IEC maturation and function (Stegmann et al. 2006). The IEC-specific knockout of mouse *Hnf4a* results in spontaneous intestinal inflammation similar to human IBD (Darsigny et al. 2009). In accord, genetic variants at human *HNF4A* are associated with risk for both UC and CD as well as colon cancer (Barrett et al. 2009; Jostins et al. 2012; Marcil et al. 2012; Chellappa et al. 2016). HNF4A is predicted to bind a majority of IBD-linked CRRs and to regulate IBD-linked genes (Haberman et al. 2014; Meddens et al. 2016). Similarly, genetic variants near human *HNF4G* have been associated with obesity and CD (Franke et al. 2007; Berndt et al. 2013). Importantly, these diverse roles for HNF4 TFs in host physiology have only been studied in animals colonized with microbiota. Therefore, the role of HNF4 in host-microbiota interactions and the implications for human IBD remain unknown.

## Results

### Hnf4a is essential for transcriptional activity from a microbiota-suppressed *cis*-regulatory DNA region

**Figure 1.**
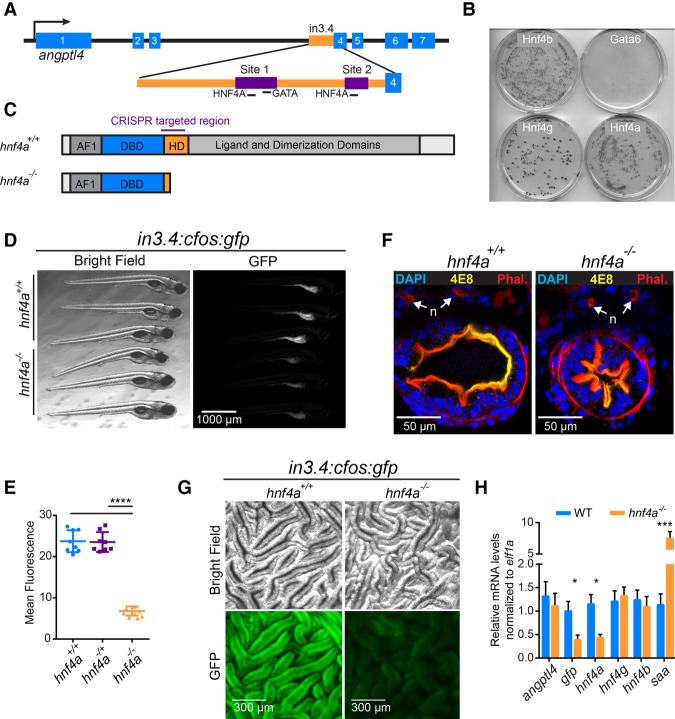
Zebrafish *hnf4a* is required for robust *in3.4:cfos:gfp* activity. (*A*) Schematic of the microbiota-suppressed zebrafish enhancer, in3.4, highlighting the regions required for intestinal activity (purple) which both contain putative HNF4 binding sites (Site 1 and Site 2) (Camp et al. 2012). (*B*) Image of four plates from the Y1H assay showing the Hnf4 family of transcription factors capable of binding in3.4 and driving expression of the antibiotic resistance reporter gene. (*C*) Hnf4a^+/+^ and Hnf4a^−/−^ protein cartoons showing the DNA binding domain (DBD) and hinge domain (HD). We characterized the two with the largest lesions, a −43 bp deletion in the hinge domain (allele designation *rdu14*) and a +25 bp insertion in the hinge domain (allele designation *rdu15*), which both result in frame-shift and early stop codons and significantly reduced transcript. (*D*) Stereofluorescence GFP and bright-field microscopy showing representative *hnf4a*^+/+^ (*top* 3) and *hnf4a*^−/−^ (*bottom* 3) 6dpf *in3.4:cfos:gfp* zebrafish. Genotype was blinded, and samples were arranged by intensity of GFP fluorescence. (*E*) GFP fluorescence (mean ± SEM) in *hnf4a*^+/+^ (*n* = 8), *hnf4a*^+/−^ (*n* = 8), and *hnf4a*^−/−^ (*n* = 8) 6-dpf *in3.4:cfos:gfp* zebrafish (Two-tailed *t*-test: *t* = 17.84, 16.51, respectively, df = 14, and [****] *P* < 0.0001). (*F*) Confocal microscopy showing representative axial cross sections in 6-dpf *hnf4a*^+/+^ (*n* = 4) and *hnf4a*^−43/−43^ (*n* = 4) larval zebrafish. 4E8 antibody (yellow) labels the intestinal brush border, DAPI (blue), and phalloidin (red), and nephros (n). (*G*) Bright-field microscopy (*top*) and stereofluorescence GFP (*bottom*) for representative *hnf4a*^+/+^ (*n* = 3) (*left*) and *hnf4a*^−/−^ (*n* = 3) (*right*) dissected intestinal folds from adult *in3.4:cfos:gfp* zebrafish. (*H*) Relative mRNA levels (mean ± SEM) in *hnf4a*^+/+^ (*n* = 3) and *hnf4a*^−/−^ (*n* = 3) adult zebrafish intestinal epithelial cell as measured by qRT-PCR. Two-tailed *t*-test: *t* = 0.93, 5.22, 6.56, 10.65, 0.75, 0.94, respectively, df = 4, and (*) *P* < 0.05, (***) *P* < 0.001. See also Supplemental Figures S1 and S2.

To identify transcriptional regulatory mechanisms underlying microbial control of host gene expression, we took advantage of a previously identified microbiota-responsive CRR termed in3.4 located within the third intron of zebrafish *angptl4* (Fig. 1A). A GFP reporter construct under control of in3.4 termed *in3.4:cfos:gfp* drives IEC-specific expression of GFP in zebrafish IECs and is suppressed by microbiota colonization, recapitulating the microbial suppression of zebrafish *angptl4* (Camp et al. 2012). However, the factor(s) that mediate microbial suppression of in3.4 were unknown. Using a yeast one-hybrid (Y1H) assay, we tested the capacity of 150 TFs expressed in the zebrafish digestive system to bind in3.4 (Supplemental Fig. S1A,B; Supplemental Table S1) and detected an interaction only with Hnf4a, Hnf4b, and Hnf4g (Fig. 1B). When either of two predicted HNF4A motifs in in3.4 are mutated, Hnf4-in3.4 interactions in the Y1H assay and intestinal GFP expression in *in3.4:cfos:gfp* zebrafish were strongly reduced (Supplemental Fig. S1C–F). Interestingly, while Gata4, Gata5, and Gata6 have predicted motifs in in3.4 (Camp et al. 2012), these TFs did not interact in the Y1H assay. This suggests that zebrafish Hnf4 TFs are capable of binding in3.4 directly and predicted HNF4A binding motifs are necessary for directing in3.4-based transcription in vitro and in the intestine.

We hypothesized that the *hnf4* transcription factor family is required to mediate microbial suppression of in3.4 activity. Although the Y1H assay demonstrated that all three zebrafish Hnf4 members are capable of binding in3.4, we concentrated our efforts on understanding the function of Hnf4a because it is the most highly conserved Hnf4 family member (Supplemental Fig. S1G; Supplemental Table S10) and has well-documented roles in intestinal physiology (San Roman et al. 2015). To that end, we generated *hnf4a* mutant zebrafish using the CRISPR/Cas9 system (Fig. 1C; Supplemental Fig. S2A–C,E). Whole-animal *Hnf4a* knockout mice die during early embryogenesis due to failure to develop a visceral endoderm (Duncan et al. 1997), but zebrafish and other fishes do not develop that extra-embryonic tissue. We found that zebrafish homozygous for a nonsense allele of *hnf4a* are viable and survive to sexual maturity (Supplemental Fig. S2D), providing new opportunities to study the roles of HNF4A in host-microbiota interactions.

To determine if Hnf4a is essential for in3.4 activity, we crossed mutant *hnf4a* alleles to the *in3.4:cfos:gfp* transgenic reporter line. GFP expression was significantly reduced in the absence of Hnf4a, suggesting that Hnf4a activates in3.4 (Fig. 1D,E,G,H). This loss of GFP expression in *hnf4a*^−/−^ mutants was not associated with overt defects in brush border development or epithelial polarity in larval stages (Fig. 1F) nor in the establishment of intestinal folds during adult stages (Fig. 1G). However, intestinal lumen of mutant larvae was reduced in size at 6 d post-fertilization (dpf) compared to WT siblings (Fig. 1F; Supplemental Fig. S2F). Together, these data indicate Hnf4a is essential for robust activity of a microbiota-suppressed CRR. Unlike *in3.4:cfos:gfp*, *angptl4* is expressed in multiple tissues and cell types (Camp et al. 2012). To determine if intestinal *angptl4* expression is dependent on Hnf4a function, we isolated RNA from IECs from *hnf4a*^+/+^ and *hnf4*^−/−^ adult *in3.4:cfos:gfp* zebrafish and performed qRT-PCR. Adult IECs (AIECs) from *hnf4a*^−/−^ have significant reductions in mRNA for *gfp* and *hnf4a* compared to *hnf4a*^+/+^ controls. However, *angptl4* expression remained unchanged in *hnf4*^−/−^ AIECs compared to WT, suggesting *angptl4* transcript levels in the adult intestine are regulated by additional mechanisms and not solely from in3.4 or Hnf4a (Fig. 1H). Transcript levels for *hnf4g* and *hnf4b* in *hnf4a*^−/−^AIEC were also unchanged. Together, these results establish that Hnf4a is required for in3.4 activity in IECs and raises the possibility that Hnf4a may have broader roles in mediating host transcriptional and physiological responses to microbiota.

### Hnf4a activates transcription of genes that are suppressed upon microbiota colonization

**Figure 2.**
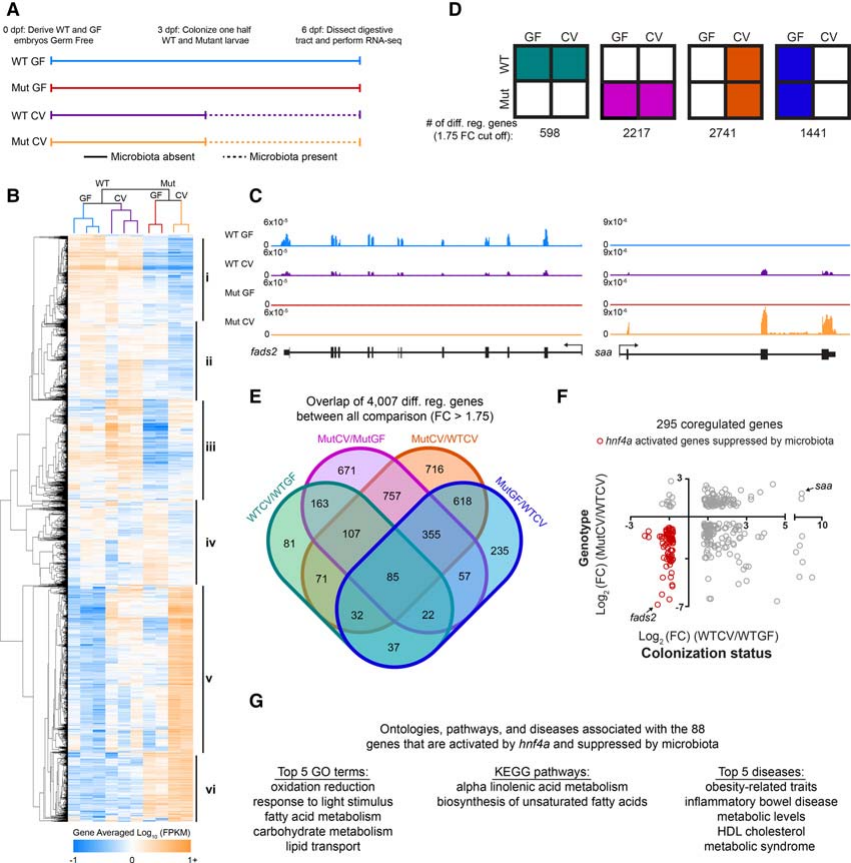
Hnf4a activates the majority of coregulated genes that are suppressed by the microbiota. (*A*) Schematic showing the experimental timeline for zebrafish digestive tract GF and CV *hnf4a*^+/+^ [WT] and *hnf4a*^−/−^ [Mut] RNA-seq experiment (*n* = 3 for WTCV and WTGF and *n* = 2 for MutCV and MutGF). (*B*) Hierarchical relatedness tree and heat map of differentially regulated genes in mutant and gnotobiotic zebrafish digestive tracts. Gene averaged log_10_ FPKMs for the biological replicates are represented for each of the 4007 differentially regulated genes. (*C*) Representative RNA-seq signal tracks at *fatty acid-desaturase 2 (fads2)* and *serum amyloid a (saa)* loci*.* (*D*) Summary of the total number of differentially expressed genes between indicated conditions (GF and CV) and genotype (WT and *hnf4a*^−/−^ [Mut]). (*E*) Four-way Venn diagram showing overlaps between all 4007 differentially regulated genes. (*F*) The 295 coregulated genes were plotted using the log_2_ (FC) calculated in the WTGF/WTCV comparison (*x*-axis) and WTCV/MutCV (*y*-axis). The 88 out of 98 genes that are activated by Hnf4a but suppressed by the microbiota are highlighted (red) and (*G*) their GO term, KEGG pathway, and disease associations are listed. See also Supplemental Figure S3.

To better define the roles of Hnf4a in microbiota response and other aspects of digestive physiology, we used RNA-seq to compare mRNA levels from digestive tracts isolated from *hnf4a*^+/+^ and *hnf4a*^−/−^ zebrafish larvae in the presence (CV) or absence of a microbiota (GF) (Fig. 2A). Consistent with our previous studies (Rawls et al. 2004; Kanther et al. 2011), comparison of wild-type zebrafish reared under CV vs. GF conditions revealed differential expression of 598 genes that were enriched for processes such as DNA replication, oxidation reduction, and response to bacterium (Fig. 2B,D; Supplemental Fig. S3; Supplemental Tables S2, S4). Strikingly, disruption of the *hnf4a* gene caused gross dysregulation of the transcriptional response to microbiota colonization, with the total number of microbiota responsive genes (CV vs. GF) increasing to 2217. Furthermore, comparison of the *hnf4a* mutant (Mut) vs. wild-type (WT) genotypes revealed differential expression of many genes in the CV condition (2741 genes) and GF condition (1441 genes) that inform a general role for Hnf4a in regulating genes in the intestinal tract (Fig. 2D,E). Principal components analysis (Supplemental Fig. S3A) and hierarchical clustering (Fig. 2B) of FPKM values indicated that the *hnf4a* genotype had a complex contribution to regulating genes involved in both responses to the microbiota and digestive physiology.

Because we found that Hnf4a activates the microbiota-suppressed intestinal CRR, in3.4, we hypothesized that this may represent a general regulatory paradigm for other microbiota-influenced CRRs and genes across the genome. When we compared the 598 genes that were microbiota responsive in wild-type digestive tracts with the 2741 genes that Hnf4a regulates in CV digestive tracts, we found these lists shared 295 genes that included *fads2* and *saa*, both of which have human orthologs that are either implicated (*FADS1/2*) or markers (*SAA*) of IBD (Fig. 2C–F; Plevy et al. 2013; Costea et al. 2014). While loss of Hnf4a could be pleiotropic, strikingly, the overlap between these subsets reveals that a disproportionate 88 of the 98 (∼90%) microbiota-suppressed genes are activated by Hnf4a (Fig. 2F; Supplemental Table S2). These 88 genes represent almost half of all 185 genes suppressed by the microbiota. These data suggest, like its role at in3.4, Hnf4a plays a critical role in directly activating a large percentage of genes that are suppressed by microbial colonization. This set of Hnf4a-activated microbiota-suppressed genes is enriched for ontologies and pathways involved in lipid and carbohydrate metabolism, suggesting microbiota might regulate these processes through suppression of Hnf4a (Fig. 2G). Interestingly, the top two diseases associated with this gene set were obesity-related traits and IBD (Fig. 2G; Supplemental Table S11). Based on these results, we hypothesized that Hnf4a DNA binding is lost upon microbial colonization within CRRs associated with microbiota-suppressed genes.

### HNF4A binding sites are enriched in promoters near genes associated with microbiota-regulated H3K27ac marks

**Figure 3.**
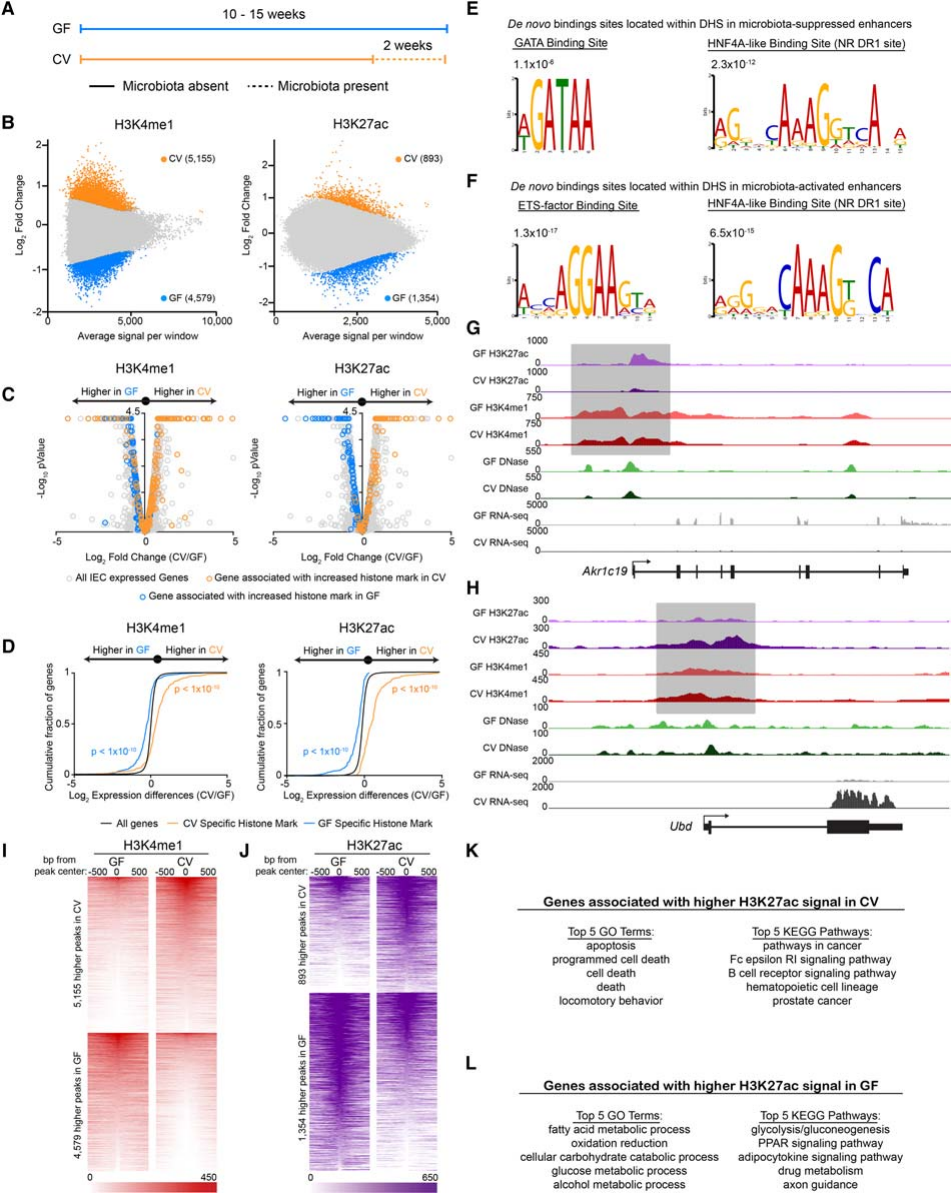
Microbiota colonization results in targeted alterations in enhancer activity near microbiota-responsive genes. (*A*) Schematic showing the gnotobiotic experimental timeline for testing mRNA levels and chromatin architecture in GF and CV mice. (*B*) MA plots from DESeq2 analysis (FDR < 0.01) of H3K4me1 (*n* = 3 per condition) (*left*) and H3K27ac (*n* = 2 per condition) (*right*) ChIP-seq from GF and CV mouse jejunal IECs. Colored dots signify regions significantly enriched for a histone mark in GF (blue) or CV (orange). We found 4579 unique H3K4me1 and 1354 unique H3K27ac peaks in GF and 5155 unique H3K4me1 and 893 unique H3K27ac peaks in CV. (*C*) Volcano plots showing pairwise comparison of RNA expression between GF (*n* = 2) and CV (*n* = 2) jejunal IECs. Blue and orange dots represent genes associated with a region enriched for H3K4me1 (*left*) or H3K27ac (*right*) signal in GF or CV. (*D*) Two-sided Kolmogorov-Smirnov goodness-of-fit test shows a positive relationship on average between the presence of a region enriched for H3K4me1/H3K27ac signal in a specific colonization state and increased transcript abundance of a neighboring gene in that same colonization state. (*E*) Top de novo binding site motifs found in DHSs that are flanked by regions enriched with H3K27ac signal in GF (*E*) or CV (*F*). Representative ChIP-seq tracks highlighting a microbiota-regulated gene associated with differential histone marks in GF (*G*) (*Akr1c19*, aldo-keto reductase family 1, member C19) or CV (*H*) (*Ubd*, ubiquitin D). Heat maps showing the average GF and CV H3K4me1 (*I*) or H3K27ac (*J*) signal at the 1000 bp flanking differential sites. (*K*,*L*) GO terms and KEGG pathways enriched in genes associated with differential H3K27ac sites shown in *J*. See also Supplemental Figure S4.

Previous attempts to identify microbial responsive enhancers genome-wide were complicated by the lack of significant changes in chromatin DNase accessibility between GF and CV IECs from mouse colon and ileum (Camp et al. 2014). These previous findings suggested other chromatin dynamics may be involved in regulating the IEC response to microbiota. We therefore sought to provide a genomic context for understanding how the microbiota alter HNF4A activity and chromatin modifications in IECs by performing RNA-seq, DNase-seq, and ChIP-seq for the enhancer histone modifications H3K4me1 and H3K27ac and the HNF4 TF family members HNF4G and HNF4A in CV and GF conditions, totaling 35 data sets. We conducted these experiments in jejunal IECs from gnotobiotic mice because (1) ChIP-grade antibodies for mouse HNF4A and HNF4G are available, (2) the relatively large organ size in mice provided sufficient numbers of IECs for ChIP-seq experiments, and (3) we speculated that the roles of HNF4A in host response to microbiota may be conserved to mammals. We first performed DNase-seq in jejunal IECs from mice reared GF or colonized for 2 wk with a conventional mouse microbiota to determine the impact of microbiota colonization on chromatin accessibility (Fig. 3A). In accord with previous studies that tested for chromatin accessibility in ileal or colonic IECs from GF or CV mice (Camp et al. 2014), we similarly found no differential DNase hypersensitivity sites (DHSs) in GF or CV jejunum (data not shown, but see Supplemental Fig. S4A–D; Supplemental Tables S6, S8). These data indicate that gross accessibility changes in chromatin do not underlie the transcription of microbiota-responsive genes in IECs.

To test if other metrics of chromatin utilization were dynamically regulated by microbiota, we performed ChIP-seq from GF and CV mouse jejunal IECs for histone marks H3K4me1 and H3K27ac that are enriched at poised enhancers and active enhancers, respectively (Fig. 3B). By determining the single-nearest gene TSS within 10 kb of the differential histone marks and overlaying these data with our new RNA-seq data sets, we found that regions that gain poised (H3K4me1) and activated (H3K27ac) enhancers upon colonization are associated with genes that have increased transcript levels upon colonization (Fig. 3C,H–K; Supplemental Fig. S4I; Supplemental Tables S3, S6, S8). Similarly, regions that lose poised and active enhancers upon colonization are associated with microbiota-suppressed genes (Fig. 3C,G,I,J,L; Supplemental Fig. S4J; Supplemental Tables S3, S6, S8). A two-sided Kolmogorov-Smirnov goodness-of-fit test shows a positive relationship between differential H3K4me1/H3K27ac regions and increased transcript abundance of nearby genes in the same colonization state (Fig. 3D). Collectively, we identified for the first time a genome-wide map of hundreds of newly identified microbial regulated CRRs, suggesting that microbiota regulation of host genes in the intestinal epithelium is mechanistically linked to histone modification changes more than gross chromatin accessibility changes (Camp et al. 2014).

We leveraged this novel atlas of microbiota-regulated enhancers and accessible chromatin to determine which TFs are predicted to bind to these regions. An unbiased analysis found that three HNF4A binding site motifs were significantly (*P* < 1 × 10^−45^, *P* < 1 × 10^−28^, and *P* < 1 × 10^−13^) enriched in promoters of genes associated with microbiota-suppressed enhancers (Supplemental Fig. S4E), and STAT1 binding site motifs were significantly (*P* < 1 × 10^−16^) enriched in promoters of genes associated with microbiota-activated enhancers (Supplemental Fig. S4F). Interestingly, DHSs associated with differentially active enhancers were enriched for two different sets of TF binding sites. DHSs flanked by microbiota-inactivated enhancers were enriched for nuclear receptor DR1 sites, which can be recognized by HNF4A (Fang et al. 2012), and GATA binding sites (*P* = 2.3 × 10^−12^ and 1.1 × 10^−6^, respectively) (Fig. 3E). DHSs associated with microbiota-activated enhancers were similarly enriched for the nuclear receptor DR1 binding sites but also for STAT/IRF-like and ETS binding sites (*P* = 6.5 × 10^−15^ and 1.3 × 10^−17^, respectively) (Fig. 3F). These data suggest that nuclear receptors like HNF4A may play a central role in IEC responses to microbial colonization.

### Microbiota colonization is associated with a reduction in HNF4A and HNF4G cistrome occupancy

**Figure 4.**
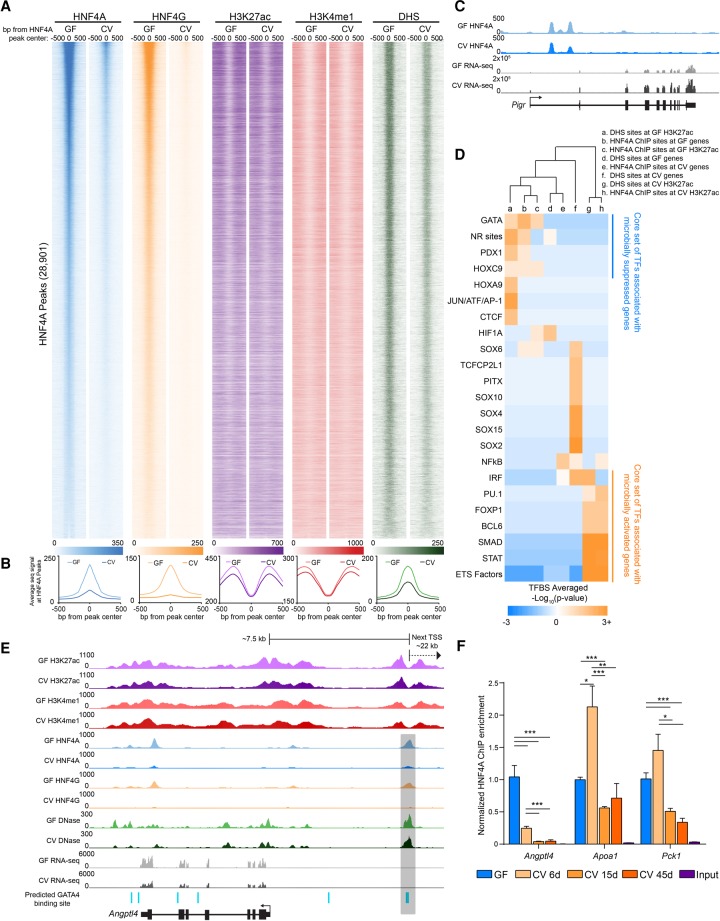
Microbiota colonization results in extensive loss of HNF4A and HNF4G DNA binding in IECs. (*A*) Heat maps showing the average GF and CV ChIP-seq or DNase-seq signal at the 1000 bp flanking HNF4A sites found in GF. (*B*) Line plots showing the average GF (light-colored line) and CV (dark-colored line) ChIP-seq and DNase-seq RPKM-normalized signal for the indicated TF, histone mark, or DHS at the 1000 bp flanking HNF4A sites found in GF (HNF4A: *n* = 3 per condition; HNF4G: *n* = 4 per condition; H3K27ac: *n* = 2 per condition; H3K4me1: *n* = 3 per condition; DNase: *n* = 3 for CV, *n* = 2 for GF). (*C*) Representative signal tracks highlighting a microbiota-induced gene (*Pigr*, polymeric immunoglobulin receptor) that is associated with an HNF4A peak with similar signal in both GF and CV jejunal IECs. (*D*) Heat map showing the enrichment of TFBS motifs within 50 bp of the DHS or HNF4A peak maxima. (*E*) Representative signal track at *Angptl4* highlighting two GATA4 sites within an HNF4A-bound region. (*F*) Bar graph showing HNF4A ChIP-PCR results at *Angptl4*, *Apoa1*, and *Pck1* loci from jejunal IECs from mice colonized for 0 (*n* = 2), 6 (*n* = 3), 15 (*n* = 2), and 45 (*n* = 3) d. Data are relative to the GF condition and normalized against a negative control locus (*Neurog1*). (*) *P* < 0.5, (**) *P* < 0.005, (***) *P* < 0.0005. See also Supplemental Figures S5 and S6.

To directly evaluate the impact of microbiota on HNF4A activity, we tested the plasticity of the genome-wide distribution of HNF4 family members in response to microbial colonization. HNF4A bound 28,901 sites and HNF4G bound 21,875 sites across the genome in GF conditions in jejunal IECs, with ∼80% of these sites being bound by both TFs. In striking contrast, the number of sites bound by HNF4A and HNF4G in CV conditions was ∼10-fold less (Fig. 4A,B; Supplemental Fig. S5A–D; Supplemental Tables S5, S8). Of the 3964 HNF4A binding sites detected in CV, there were only 267 HNF4A sites that were specific to the CV condition (Supplemental Fig. S6A,C; Supplemental Table S8). Yet, the genes associated with these HNF4A sites that are retained in CV are enriched for ontologies and pathways fundamental to intestinal epithelial biology (Supplemental Fig. S6B). Surprisingly, we found that HNF4A sites are equally distributed between genes that are up-regulated in both GF and CV conditions (Supplemental Fig. S6E). However, we did find that the average CV HNF4A ChIP-seq signal strength was significantly increased at HNF4A sites associated with microbiota-induced genes relative to those HNF4A sites associated with microbiota-suppressed genes, suggesting HNF4A may play a limited role in genes up-regulated by colonization (Supplemental Fig. S6F). In contrast, GF HNF4A ChIP-seq signal was equivalent at HNF4A sites associated with microbiota-suppressed and induced genes (Supplemental Fig. S6F). Interestingly, we found that HNF4A sites correspond with increased H3K27ac, H3K4me1, and DHS signal in GF compared to these same chromatin marks in CV (Supplemental Fig. S6G). We do not believe that the reduction of HNF4A binding is the result of chromatin quality in a particular condition since there are genomic locations where GF and CV HNF4A sites appeared to have equivalent signal (Fig. 4C). Furthermore, ChIP enrichment in these IEC preparations for another zinc finger TF, CTCF, was unaffected by microbiota colonization (Supplemental Fig. S6D). This indicates that the observed reduction of HNF4 ChIP-seq signal in CV IECs is a result of microbiota on HNF4 binding and is not the result of altered ChIP efficiency or sample quality in the different conditions. To test if microbial suppression of HNF4A occupancy is persistent, we performed ChIP-PCR from ex-GF mice that were colonized with microbiota for 6, 15, or 45 d. We found that even after 45 d post-colonization, HNF4A occupancy at binding sites was significantly reduced compared to GF (Fig. 4F). The dramatic loss of HNF4A and HNF4G DNA binding upon colonization is consistent with HNF4A acting as a potent activator of microbiota-suppressed genes.

We further speculated that certain coregulatory sequence-specific transcription factors may also contribute to regulating transcription with HNF4 at these sites. To explore this possibility, we searched for TF motifs associated with HNF4A ChIP sites and found an enrichment of putative binding sites for TFs known to be involved in small intestinal physiology (GATA and HOXC9) as well as nutrient metabolism (PDX1) at both HNF4A-bound regions associated with genes and enhancers suppressed by microbes (Fig. 4D). We similarly found GATA sites located within an HNF4A-bound CRR near murine *Angptl4* (Fig. 4E), similar to the coincident HNF4 and GATA motifs in zebrafish in3.4 (Camp et al. 2012). Furthermore, binding sites for TFs known to be involved in cell proliferation and cell death (ETS transcription factor family) are enriched near HNF4A bound regions that intersect microbiota-induced enhancers (Fig. 4D). Collectively, our integrative analyses of these novel ChIP-seq, DNase-seq, and RNA-seq data sets identify a core set of putative microbiota-responsive TFs that may interact with HNF4A to mediate microbial control of IEC gene expression. These results suggest HNF4A plays a major role in integrating microbial signals to regulate gene expression and raise the possibility that this novel microbiota-HNF4A axis might contribute to human disease.

### Microbiota-mediated suppression of HNF4A may contribute to gene expression profiles associated with human IBD

**Figure 5.**
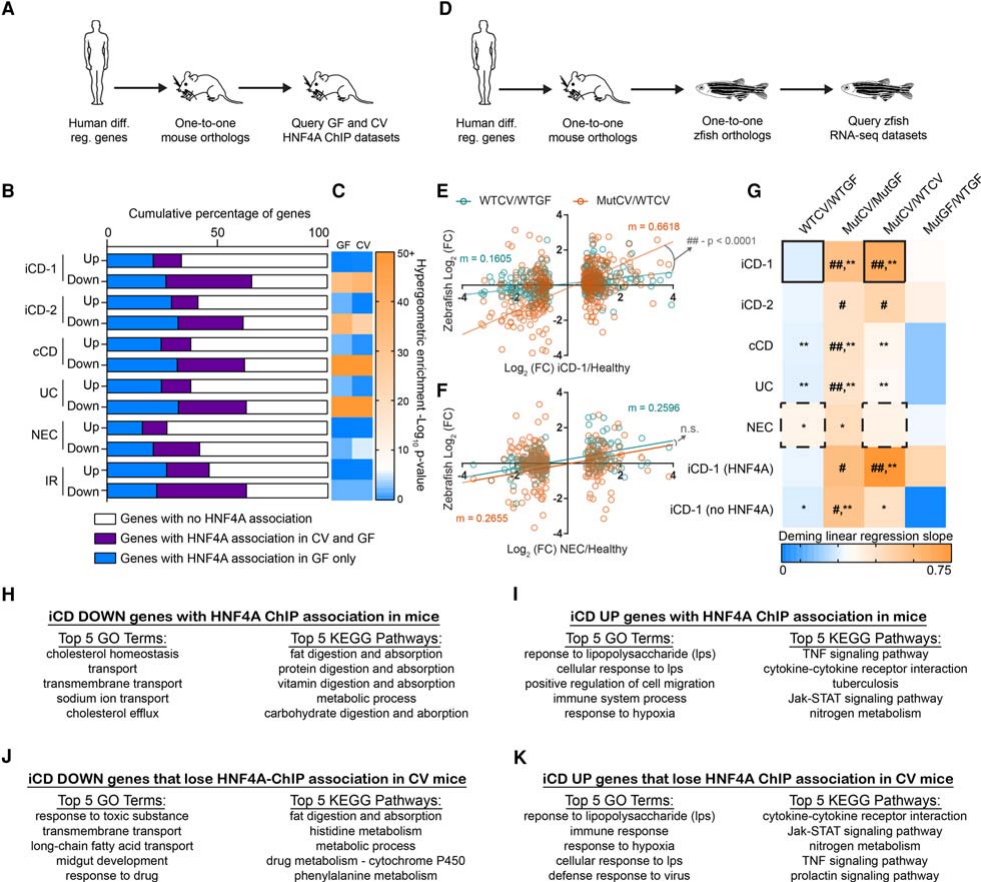
Microbiota suppression of HNF4A activity is highly correlated with genes and intestinal processes suppressed in human IBD and conserved in zebrafish. (*A*) Flow chart showing the experimental design and filters used to identify IBD, NEC, or IR gene orthologs associated with mouse HNF4A ChIP sites. (*B*) Bar chart showing the proportion of HNF4A associations in GF and CV mouse jejunal IECs near human-to-mouse one-to-one gene orthologs differentially regulated in human pediatric ileal Crohn's disease (iCD-1), adult iCD (iCD-2), adult colonic Crohn's disease (cCD), adult ulcerative colitis (UC), neonatal necrotizing enterocolitis (NEC), or insulin-resistance (IR). (*C*) Heat map representing the −log_10_ (*P*-value) of the enrichment of GF or CV HNF4A-associated genes that are differentially regulated genes in the indicated IBD data sets. Log_10_ *P*-values were calculated using a hypergeometric enrichment analysis and converting all HNF4A ChIP associated mouse genes to human orthologs (GF = 5863 genes and CV = 2119 genes). (*D*) Flow chart showing the experimental design and filters used to identify correlations between gnotobiotic WT or mutant zebrafish gene expression and gene orthologs differentially expressed in human IBD or NEC. Example of Deming linear regression analysis showing the correlation of log_2_ (FC) between WTCV/WTGF (*E*) or MutCV/WTCV (*F*) zebrafish and pediatric iCD or NEC. *m* = slope of the line. (*G*) Heat map representing slopes of Deming linear regression lines showing positive correlative relationships between the log_2_ gene expression fold changes of one-to-one orthologs from human diseases compared to log_2_ fold changes in zebrafish WTCV/WTGF, MutCV/MutGF, MutCV/WTCV, and MutGF/WTGF. Because loss of *hnf4a* function in zebrafish appeared to resemble more closely the iCD signature than cCD or UC, we performed pairwise comparisons of gene orthologs that are (1) differentially regulated in human iCD and (2) have a mouse HNF4A ChIP association (iCD-1 [HNF4A]) or do not have a mouse HNF4A ChIP association (iCD-1 [no HNF4A]). Hash signs indicate slope of Deming linear regression lines is significantly greater than WTCV/WTGF comparison. (#) *P* < 0.05, (##) *P* < 0.0001. Asterisks indicate slope of Deming linear regression line is significantly greater than MutGF/WTGF. (*) *P* < 0.05, (**) *P* < 0.001. Solid boxes correspond to slope of lines in panel *D*, and dashed boxes correspond to slope of lines in panel *E*. (*H*–*K*) The top five GO terms and the top five KEGG pathways for indicated gene lists.

Both HNF4A and the intestinal microbiota have been separately implicated in the pathogenesis of the human IBDs Crohn's disease and ulcerative colitis (Ahn et al. 2008; Sartor and Wu 2016). However, a mechanistic link between microbiota and HNF4A in the context of IBD pathogenesis has not been established. Previous transcriptomic studies have identified genes differentially expressed in ileal (iCD) and colonic CD (cCD) and UC (Arijs et al. 2009; Haberman et al. 2014) biopsies. We queried these human gene lists to identify one-to-one orthologs in mice and referenced them against our new gnotobiotic mouse jejunal HNF4A ChIP-seq data (Fig. 5A). Strikingly, the majority of human genes down-regulated in each of these IBD data sets have mouse orthologs that are associated with an HNF4A-bound region (Fig. 5B,C; Supplemental Table S7). Focusing on the iCD data set from the largest of these previous studies (Haberman et al. 2014), we found that differential iCD genes associated with HNF4A sites are enriched for distinct ontologies and pathways that are dysregulated in IBD (Fig. 5H–K). In contrast to IBD, analysis of intestinal transcriptomic data sets from human subjects with necrotizing enterocolitis (NEC) (Tremblay et al. 2016) or insulin-resistance (IR) (Veilleux et al. 2015) did not reveal strong enrichment of HNF4A-bound regions near down-regulated genes (Fig. 5C). Notably, in each of these CD, UC, NEC, and IR data sets, a greater percentage of down-regulated genes were linked to HNFA-bound regions compared to up-regulated genes (Fig. 5B). These data suggest that microbiota-dependent and microbiota-independent suppression of HNF4A activity in the intestine might play an important role in IBD pathologies. To assess if microbiota suppression of HNF4A activity regulates genes differentially expressed in IBD, we queried the published human IBD and NEC gene expression data sets to identify human-mouse-zebrafish one-to-one-to-one orthologs that were differentially expressed in our RNA-seq analysis of gnotobiotic zebrafish *hnf4a* mutants (Fig. 5D). We found that ortholog expression fold changes in human IBD/healthy comparisons most closely resembled the expression fold changes of MutCV/MutGF and MutCV/WTCV (Fig. 5E–G). Neither the WTCV/WTGF nor the MutGF/WTGF comparisons faithfully recapitulate the expression profiles of IBD/healthy comparisons. These data indicate that microbiota colonization in the absence of *hnf4a* function in zebrafish is sufficient to induce a gene expression profile that resembles human IBD. Strikingly, the positive correlation and significant resemblance to the iCD-like gene signatures in the colonized *hnf4a*^−/−^ compared to colonized *hnf4a*^+/+^ zebrafish digestive tracts become even stronger when we limited our analysis to one-to-one orthologs that have an association with an HNF4A-bound region in mouse IECs (Fig. 5G). Together, these results indicate that intestinal suppression of HNF4A target genes is a prevalent feature of human CD and UC and suggest a model wherein HNF4A maintains transcriptional homeostasis in the presence of a microbiota and protects against an evolutionarily conserved IBD-like gene expression signature.

## Discussion

Over the course of animal evolution, the intestinal epithelium has served as the primary barrier between animal hosts and the complex microbial communities they harbor. IECs maintain this barrier and perform their physiological roles in nutrient transport and metabolism through dynamic transcriptional programs. The regulatory mechanisms that orchestrate these transcriptional programs represent potential therapeutic targets for a variety of human intestinal diseases, including IBD. Here, we discovered that HNF4A activity and its transcriptional network are suppressed by microbiota. HNF4 is the oldest member of the nuclear receptor TF family (Bridgham et al. 2010), and our findings in fish and mammals suggest that microbial suppression of HNF4A may be a conserved feature of IEC transcriptional programs present in the common ancestor.

We discovered HNF4A as a microbiota-suppressed transcription factor by demonstrating that it specifically binds to a microbiota-suppressed *cis*-regulatory element, in3.4, which is located at the zebrafish gene *angptl4*. This finding, combined with our zebrafish RNA-seq data (Fig. 2F,G), revealed a broad role for HNF4A in activation of microbially suppressed transcripts. Though *hnf4a* mutant zebrafish have reduced in3.4 activity in the intestinal epithelium based on transgenic reporter assays, the transcript levels of the endogenous zebrafish *angptl4* gene appears unaffected in both larval digestive tracts and adult IECs. The zebrafish genome encodes two additional HNF4 family members (*hnf4b*, *hnf4g*), and previous studies in mammals have shown *Angptl4* can be regulated by other metabolically activated nuclear receptors (Staiger et al. 2009; Korecka et al. 2013). We hypothesize that loss of HNF4A function may lead to a metabolic imbalance leading to atypical or compensatory activation of other *trans*- and *cis*-factors that control expression of *angptl4* and other genes in the intestine.

Our results suggest new links between HNF4A and microbiota in the context of human IBD. IBD patients, particularly those suffering from Crohn's disease, often present with decreased serum low-density lipoprotein levels and reduced total cholesterol levels compared to healthy individuals (Hrabovsky et al. 2009; Agouridis et al. 2011). These serum levels are consistent with reduced transcript levels for genes involved in intestinal absorption and transport of lipid and cholesterol in ileal and colonic biopsies from UC and CD patients (Arijs et al. 2009; Haberman et al. 2014). Transcription factors, including nuclear receptors like HNF4A and FXR, are known to regulate bile acid production and lipid and cholesterol absorption and have already been implicated in IBD (Ahn et al. 2008; Nijmeijer et al. 2011). Previous studies have shown that some IBD-associated H3K27ac-activated regions that also overlap with IBD-associated SNPs contain HNF4A binding sites (Mokry et al. 2014). This is consistent with our findings and supports a role for HNF4A in regulating gene expression and inflammation in the context of IBD. However, our work is the first to demonstrate a role for microbiota in suppressing HNF4A and to implicate microbiota-HNF4A interactions in driving an IBD-like gene expression signature (Fig. 5). In addition to IBD, human *HNF4A* variants are associated with metabolic syndrome (Weissglas-Volkov et al. 2006) and type 2 diabetes (Ma et al. 2016). Interestingly, microbiota have also been implicated in both of these diseases (Qin et al. 2012; Vrieze et al. 2012), raising the possibility that microbiota suppression of HNF4A *trans* activity could play a role in these diseases as well. Indeed, we find that genes down-regulated in intestinal tissue from IR-obese patients have increased HNF4A binding associations compared to up-regulated genes (Veilleux et al. 2015), similar to the enrichment of HNF4A associations at down-regulated genes in IBD (Fig. 5B,C). Interestingly, up-regulated genes in these IR-obese patients were enriched for pro-inflammatory markers. This underscores the relationship between metabolic impairments and inflammation in the intestine and prompts further investigation of how HNF4A might contribute to this relationship. HNF4A has been shown to play key roles in anti-oxidative and anti-inflammatory defense mechanisms (Marcil et al. 2010), so aberrant microbial suppression could promote an inflammatory state. HNF4A target genes are down-regulated in human IBD (Arijs et al. 2009; Haberman et al. 2014) and mouse experimental colitis (Chahar et al. 2014), and the HNF4A target *APOA1* has been shown to be protective against intestinal inflammation in mice (Gkouskou et al. 2016). We speculate that the genes governed by this novel microbiota–HNF4A axis may include additional anti- and pro-inflammatory factors that could provide new targets for IBD therapy.

Our results reveal similar effects of microbiota colonization and experimental colitis on HNF4A cistrome occupancy in the intestine, but the underlying molecular mechanisms are unresolved. DSS-induced colitis results in reduced HNF4A protein levels and altered cellular localization (Chahar et al. 2014); however, our results indicate the microbiota neither reduce HNF4A protein levels nor impact its nuclear localization in jejunal IECs 2 wk after colonization (Supplemental Fig. S6H,I). Colonization of GF mice with microbiota initiates a transcriptional adaptation in the intestine that progresses for several weeks before reaching homeostasis (El Aidy et al. 2012). However, our data indicate HNF4A suppression is achieved within 15 d and persists through at least 45 d after colonization. These data collectively suggest that microbiota suppress HNF4A activity in the jejunum through mechanisms distinct from those utilized in DSS-induced colitis.

HNF4A has been characterized as a master metabolic regulator for its conserved roles in gluconeogenesis, glucose homeostasis, and fatty acid metabolism (Palanker et al. 2009; Frochot et al. 2012; Barry and Thummel 2016). Despite its clear importance in metabolic health, relatively little insight into its regulation in a biological context has been reported. In vitro and cell culture studies have identified possible suppressors and activators of HNF4A, including acetylation by CREB-binding protein (CREBBP, also known as CBP), which has been shown to induce HNF4A activity (Soutoglou et al. 2000; Hong et al. 2003). The nuclear receptor cofactor and master regulator of mitochondrial biogenesis PPARGC1A binds HNF4A and promotes activation of HNF4A target genes (Rha et al. 2009). Colonization of GF animals with microbiota leads to increased energy harvest (Rabot et al. 2010; Semova et al. 2012) and changes in metabolic homeostasis including decreased AMPK complex activity in skeletal muscle and liver (Backhed et al. 2007). Previous studies have also shown that the activated AMPK complex phosphorylates and activates PPARGC1A (Jager et al. 2007); therefore, microbiota might suppress HNF4A activity indirectly through induced alterations in metabolic homeostasis. Other studies have shown that HNF4A activity is controlled through use of alternative promoters which generate different isoforms (Huang et al. 2009). However, we did not detect differential *Hnf4a* exon usage by DEXseq (Li et al. 2015) in our RNA-seq data from GF and CV IECs (data not shown). Another facet of HNF4A biology that remains unresolved is the identity of its endogenous ligand(s). Although historically considered an orphan nuclear receptor, several fatty acids (FA), including linoleic acid, have been identified as ligands for HNF4A (Hertz et al. 1998; Palanker et al. 2009; Yuan et al. 2009). Fatty acids are an attractive class of putative regulators of HNF4A, since the microbiota are known to regulate FA absorption in zebrafish IECs (Semova et al. 2012). Further, specific bacterial taxa are known to modify the structure of polyunsaturated FAs (PUFAs), and these native and modified PUFAs have distinct impacts on animal health (O'Shea et al. 2012) and may serve as therapeutics for IBD (Mbodji et al. 2013).

In our attempt to understand how the microbiota regulate HNF4A activity and host gene transcription, we were motivated to investigate if microbiota impact histone modification and chromatin accessibility in the mouse jejunum. Our findings support the model that microbiota alter IEC gene expression by affecting TF binding and histone modification at tissue-defined open chromatin sites (Camp et al. 2014). We provide the genomic addresses of hundreds of microbiota-regulated enhancers as well as the genes associated with these enhancers and HNF4A binding sites. Similar to other findings in intra-epithelial lymphocytes (Semenkovich et al. 2016), our work demonstrates a clear microbial contribution to the modification of the histone landscape in IECs and provides another important layer of regulation that orchestrates microbiota regulation of host genes involved in intestinal physiology and human disease. We were also able to establish a link between microbiota-regulated genes and enhancers and NR binding sites. These NR binding sites are coincident with a core set of TFs that are enriched near microbiota-suppressed enhancers/genes (GATA) or induced enhancers/genes (ETS-factors and IRF) (Supplemental Fig. S7). GATA4 was previously shown to be a positive regulator of genes suppressed by microbiota in the mouse jejunum (Shulzhenko et al. 2011), supporting potential coregulatory interactions with HNF4A. Coregulation by other TFs represents one possible mode of HNF4A regulation by which the microbiota could suppress HNF4A activity without impacting the gene transcription of all HNF4A-associated genes.

## Methods

### Yeast one-hybrid ORFeome screen

The yeast one-hybrid ORFeome screen was performed using the Clontech Matchmaker Gold Yeast One-hybrid Library Screening System (cat. #630491) protocol with the following exceptions: The Y1HGold yeast strain was transformed using standard yeast transformation procedures with BstBI-digested pBait-AbAi containing either the WT or a SDM in3.4 or the TP53 binding site sequence, and positive transformants were selected on SD/−URA media. In addition, a ORFeome library consisting of 148 zebrafish transcription factors cloned from adult zebrafish liver (Supplemental Table S1) plus *hnf4a* and *hnf4g* cDNAs in pDEST22 prey vectors containing an N-terminal GAL4-activation domain were utilized (Boyle et al. 2017). For additional information, see Supplemental Methods.

### Mouse IEC isolation for DNase-, ChIP-, and RNA-seq

The small intestine was removed from the mouse, and the jejunum was excised from the duodenum and ileum. Duodenum was defined as the anterior 5 cm of the midgut, and ileum was defined as posterior 6 cm of midgut as described (Camp et al. 2014). Adipose and vasculature were removed from the tissue. The jejunum was opened longitudinally along the length of the tissue, exposing the lumen and epithelial cell layer. Luminal debris was washed away from the epithelia with ice-cold sterile PBS. The tissue was temporarily stored in 10 mL of ice-cold sterile PBS with 1× protease inhibitors (cOmplete EDTA-Free, Roche, #11873580001) and 10 µM Y-27632 (ROCK I inhibitor, Selleck Chemicals, #S1049) to inhibit spontaneous apoptosis. The jejunum was moved into a 15-mL conical tube containing 3 mM EDTA in PBS with 1× protease inhibitors and 10 µM Y-27632. The tissue was placed on a nutator in a cold room for 15 min. The jejunum was removed from the 3 mM EDTA and placed on an ice-cold glass Petri dish with PBS containing 1 mM MgCl_2_ and 2 mM CaCl_2_ with protease inhibitors and 10 µM Y-27632. Villi were scraped off of the tissue using the side of a sterile plastic micropipette and transferred into a new 15-mL conical tube. The isolated IECs were then crosslinked for ChIP-seq, ChIP-PCR or used for DNase-seq or RNA-seq. For additional information, see Supplemental Methods.

### Bioinformatic and statistical analysis

Sample sizes for zebrafish experiments (noted in figure legends) were selected based on genotype availability and transgenesis efficiency. All sample collection was performed two or more times on independent days. For sequencing experiments, statistical calls for differential gene expression were made by Cuffdiff2 (Trapnell et al. 2013). For the zebrafish RNA-seq experiment, next-generation sequencing was performed once and at the same time to avoid batch effects: WTGF and WTCV (*n* = 3); MutGF and MutCV (*n* = 2). We originally collected *n* = 3 MutGF and MutCV biological replicates; however, using pre-established criteria and to avoid RNA contamination, we excluded one biological replicate from all analysis from these groups because of sequencing reads that mapped within the deleted *hnf4a* exon in the *hnf4a*^−/−^ genotype.

GF mice were randomly chosen by gnotobiotic staff for microbiota colonization (CV) based on their availability and litter sizes. All sample collection was performed two or more times per condition on independent days. GF and CV mouse samples were collected on different days. For sequencing experiments, statistical calls for differential gene expression and differential peak calls were made by Cuffdiff2, MACS2, and DESeq2 (Zhang et al. 2008; Anders and Huber 2010; Trapnell et al. 2013; Love et al. 2014). For the mouse RNA-seq experiment, next-generation sequencing was performed once and at the same time to avoid batch effects: GF (*n* = 2) and CV (*n* = 2). Paired GF and CV ChIP and library amplification was performed simultaneously. Typically, biological ChIP replicates were sequenced on different days and were always paired with the other condition (i.e., CV and GF were always sequenced together). The number of biological ChIP replicates (noted in figure legends) was dependent on reproducibility between ChIP samples and/or our ability to determine statistical differential sites using DESeq2 (for H3K4me1 and H3K27ac).

All statistical metrics (except where otherwise noted) were performed in Graphpad Prism 7.01. Deming linear regression was used for Figure 5 because it is a stronger and more accurate assessment of correlation when both the *x* and *y* variables have experimental error. Details regarding the other statistical tests used in this study can be found in the figure legends or above.

For detailed methods on animal husbandry, zebrafish transgenesis, zebrafish mutagenesis, imaging, immunostaining, site-directed mutagenesis, and ChIP-, RNA-, and DNase-seq preparation and analysis, please see Supplemental Methods.

## Data access

Transcription factor ChIP-seq, Histone ChIP-seq, DNase-seq, and RNA-seq data sets from this study have been submitted to the NCBI Gene Expression Omnibus (GEO; http://www.ncbi.nlm.nih.gov/geo/) under accession number GSE90462.

## Supplementary Material

Supplemental Material

## References

[DAVISONGR220111C1] Agouridis AP, Elisaf M, Milionis HJ. 2011 An overview of lipid abnormalities in patients with inflammatory bowel disease. Ann Gastroenterol 24: 181–187. 24713706 PMC3959314

[DAVISONGR220111C2] Ahn SH, Shah YM, Inoue J, Morimura K, Kim I, Yim S, Lambert G, Kurotani R, Nagashima K, Gonzalez FJ, 2008 Hepatocyte nuclear factor 4α in the intestinal epithelial cells protects against inflammatory bowel disease. Inflamm Bowel Dis 14: 908–920. 18338782 10.1002/ibd.20413 PMC2435391

[DAVISONGR220111C3] Alenghat T, Osborne LC, Saenz SA, Kobuley D, Ziegler CG, Mullican SE, Choi I, Grunberg S, Sinha R, Wynosky-Dolfi M, 2013 Histone deacetylase 3 coordinates commensal-bacteria-dependent intestinal homeostasis. Nature 504: 153–157. 24185009 10.1038/nature12687 PMC3949438

[DAVISONGR220111C4] Anders S, Huber W. 2010 Differential expression analysis for sequence count data. Genome Biol 11: R106. 20979621 10.1186/gb-2010-11-10-r106 PMC3218662

[DAVISONGR220111C5] Arijs I, De Hertogh G, Lemaire K, Quintens R, Van Lommel L, Van Steen K, Leemans P, Cleynen I, Van Assche G, Vermeire S, 2009 Mucosal gene expression of antimicrobial peptides in inflammatory bowel disease before and after first infliximab treatment. PLoS One 4: e7984. 19956723 10.1371/journal.pone.0007984 PMC2776509

[DAVISONGR220111C6] Bäckhed F, Ding H, Wang T, Hooper LV, Koh GY, Nagy A, Semenkovich CF, Gordon JI. 2004 The gut microbiota as an environmental factor that regulates fat storage. Proc Natl Acad Sci 101: 15718–15723. 15505215 10.1073/pnas.0407076101 PMC524219

[DAVISONGR220111C7] Backhed F, Manchester JK, Semenkovich CF, Gordon JI. 2007 Mechanisms underlying the resistance to diet-induced obesity in germ-free mice. Proc Natl Acad Sci 104: 979–984. 17210919 10.1073/pnas.0605374104 PMC1764762

[DAVISONGR220111C8] Barrett JC, Lee JC, Lees CW, Prescott NJ, Anderson CA, Phillips A, Wesley E, Parnell K, Zhang H, Drummond H, 2009 Genome-wide association study of ulcerative colitis identifies three new susceptibility loci, including the HNF4A region. Nat Genet 41: 1330–1334. 19915572 10.1038/ng.483 PMC2812019

[DAVISONGR220111C9] Barry WE, Thummel CS. 2016 The *Drosophila* HNF4 nuclear receptor promotes glucose-stimulated insulin secretion and mitochondrial function in adults. eLife 5: e11183. 27185732 10.7554/eLife.11183 PMC4869932

[DAVISONGR220111C10] Bates JM, Akerlund J, Mittge E, Guillemin K. 2007 Intestinal alkaline phosphatase detoxifies lipopolysaccharide and prevents inflammation in zebrafish in response to the gut microbiota. Cell Host Microbe 2: 371–382. 18078689 10.1016/j.chom.2007.10.010 PMC2730374

[DAVISONGR220111C11] Berndt SI, Gustafsson S, Magi R, Ganna A, Wheeler E, Feitosa MF, Justice AE, Monda KL, Croteau-Chonka DC, Day FR, 2013 Genome-wide meta-analysis identifies 11 new loci for anthropometric traits and provides insights into genetic architecture. Nat Genet 45: 501–512. 23563607 10.1038/ng.2606 PMC3973018

[DAVISONGR220111C12] Boyle G, Richter K, Priest HD, Traver D, Mockler TC, Chang JT, Kay SA, Breton G. 2017 Comparative analysis of vertebrate diurnal/circadian transcriptomes. PLoS One 12: e0169923. 28076377 10.1371/journal.pone.0169923 PMC5226840

[DAVISONGR220111C13] Bridgham JT, Eick GN, Larroux C, Deshpande K, Harms MJ, Gauthier ME, Ortlund EA, Degnan BM, Thornton JW. 2010 Protein evolution by molecular tinkering: diversification of the nuclear receptor superfamily from a ligand-dependent ancestor. PLoS Biol 8: e1000497. 20957188 10.1371/journal.pbio.1000497 PMC2950128

[DAVISONGR220111C14] Camp JG, Jazwa AL, Trent CM, Rawls JF. 2012 Intronic *cis*-regulatory modules mediate tissue-specific and microbial control of angptl4/fiaf transcription. PLoS Genet 8: e1002585. 22479192 10.1371/journal.pgen.1002585 PMC3315460

[DAVISONGR220111C15] Camp JG, Frank CL, Lickwar CR, Guturu H, Rube T, Wenger AM, Chen J, Bejerano G, Crawford GE, Rawls JF. 2014 Microbiota modulate transcription in the intestinal epithelium without remodeling the accessible chromatin landscape. Genome Res 24: 1504–1516. 24963153 10.1101/gr.165845.113 PMC4158762

[DAVISONGR220111C16] Chahar S, Gandhi V, Yu S, Desai K, Cowper-Sal-lari R, Kim Y, Perekatt AO, Kumar N, Thackray JK, Musolf A, 2014 Chromatin profiling reveals regulatory network shifts and a protective role for hepatocyte nuclear factor 4α during colitis. Mol Cell Biol 34: 3291–3304. 24980432 10.1128/MCB.00349-14 PMC4135557

[DAVISONGR220111C17] Chellappa K, Deol P, Evans JR, Vuong LM, Chen G, Briancon N, Bolotin E, Lytle C, Nair MG, Sladek FM. 2016 Opposing roles of nuclear receptor HNF4α isoforms in colitis and colitis-associated colon cancer. eLife 5: e10903. 27166517 10.7554/eLife.10903 PMC4907689

[DAVISONGR220111C18] Costea I, Mack DR, Lemaitre RN, Israel D, Marcil V, Ahmad A, Amre DK. 2014 Interactions between the dietary polyunsaturated fatty acid ratio and genetic factors determine susceptibility to pediatric Crohn's disease. Gastroenterology 146: 929–931. 24406470 10.1053/j.gastro.2013.12.034

[DAVISONGR220111C19] Creyghton MP, Cheng AW, Welstead GG, Kooistra T, Carey BW, Steine EJ, Hanna J, Lodato MA, Frampton GM, Sharp PA, 2010 Histone H3K27ac separates active from poised enhancers and predicts developmental state. Proc Natl Acad Sci 107: 21931–21936. 21106759 10.1073/pnas.1016071107 PMC3003124

[DAVISONGR220111C20] Darsigny M, Babeu JP, Dupuis AA, Furth EE, Seidman EG, Levy E, Verdu EF, Gendron FP, Boudreau F. 2009 Loss of hepatocyte-nuclear-factor-4α affects colonic ion transport and causes chronic inflammation resembling inflammatory bowel disease in mice. PLoS One 4: e7609. 19898610 10.1371/journal.pone.0007609 PMC2764139

[DAVISONGR220111C21] Duncan SA, Nagy A, Chan W. 1997 Murine gastrulation requires HNF-4 regulated gene expression in the visceral endoderm: tetraploid rescue of Hnf-4(−/−) embryos. Development 124: 279–287. 9053305 10.1242/dev.124.2.279

[DAVISONGR220111C22] El Aidy S, van Baarlen P, Derrien M, Lindenbergh-Kortleve DJ, Hooiveld G, Levenez F, Dore J, Dekker J, Samsom JN, Nieuwenhuis EE, 2012 Temporal and spatial interplay of microbiota and intestinal mucosa drive establishment of immune homeostasis in conventionalized mice. Mucosal Immunol 5: 567–579. 22617837 10.1038/mi.2012.32

[DAVISONGR220111C23] Evans RM, Mangelsdorf DJ. 2014 Nuclear receptors, RXR, and the Big Bang. Cell 157: 255–266. 24679540 10.1016/j.cell.2014.03.012 PMC4029515

[DAVISONGR220111C24] Fang B, Mane-Padros D, Bolotin E, Jiang T, Sladek FM. 2012 Identification of a binding motif specific to HNF4 by comparative analysis of multiple nuclear receptors. Nucleic Acids Res 40: 5343–5356. 22383578 10.1093/nar/gks190 PMC3384313

[DAVISONGR220111C25] Franke A, Hampe J, Rosenstiel P, Becker C, Wagner F, Hasler R, Little RD, Huse K, Ruether A, Balschun T, 2007 Systematic association mapping identifies NELL1 as a novel IBD disease gene. PLoS One 2: e691. 17684544 10.1371/journal.pone.0000691 PMC1933598

[DAVISONGR220111C26] Frochot V, Alqub M, Cattin AL, Carriere V, Houllier A, Baraille F, Barbot L, Saint-Just S, Ribeiro A, Lacasa M, 2012 The transcription factor HNF-4α: a key factor of the intestinal uptake of fatty acids in mouse. Am J Physiol Gastrointest Liver Physiol 302: G1253–G1263. 22461026 10.1152/ajpgi.00329.2011

[DAVISONGR220111C27] Gkouskou KK, Ioannou M, Pavlopoulos GA, Georgila K, Siganou A, Nikolaidis G, Kanellis DC, Moore S, Papadakis KA, Kardassis D, 2016 Apolipoprotein A-I inhibits experimental colitis and colitis-propelled carcinogenesis. Oncogene 35: 2496–2505. 26279300 10.1038/onc.2015.307

[DAVISONGR220111C28] Haberman Y, Tickle TL, Dexheimer PJ, Kim MO, Tang D, Karns R, Baldassano RN, Noe JD, Rosh J, Markowitz J, 2014 Pediatric Crohn disease patients exhibit specific ileal transcriptome and microbiome signature. J Clin Invest 124: 3617–3633. 25003194 10.1172/JCI75436 PMC4109533

[DAVISONGR220111C29] Hertz R, Magenheim J, Berman I, Bar-Tana J. 1998 Fatty acyl-CoA thioesters are ligands of hepatic nuclear factor-4α. Nature 392: 512–516. 9548258 10.1038/33185

[DAVISONGR220111C30] Hong YH, Varanasi US, Yang W, Leff T. 2003 AMP-activated protein kinase regulates HNF4α transcriptional activity by inhibiting dimer formation and decreasing protein stability. J Biol Chem 278: 27495–27501. 12740371 10.1074/jbc.M304112200

[DAVISONGR220111C31] Hrabovsky V, Zadak Z, Blaha V, Hyspler R, Karlik T, Martinek A, Mendlova A. 2009 Cholesterol metabolism in active Crohn's disease. Wien Klin Wochenschr 121: 270–275. 19562284 10.1007/s00508-009-1150-6

[DAVISONGR220111C32] Huang J, Levitsky LL, Rhoads DB. 2009 Novel P2 promoter-derived HNF4α isoforms with different N-terminus generated by alternate exon insertion. Exp Cell Res 315: 1200–1211. 19353766 10.1016/j.yexcr.2009.01.004

[DAVISONGR220111C33] Jager S, Handschin C, St-Pierre J, Spiegelman BM. 2007 AMP-activated protein kinase (AMPK) action in skeletal muscle via direct phosphorylation of PGC-1α. Proc Natl Acad Sci 104: 12017–12022. 17609368 10.1073/pnas.0705070104 PMC1924552

[DAVISONGR220111C34] Jostins L, Ripke S, Weersma RK, Duerr RH, McGovern DP, Hui KY, Lee JC, Schumm LP, Sharma Y, Anderson CA, 2012 Host-microbe interactions have shaped the genetic architecture of inflammatory bowel disease. Nature 491: 119–124. 23128233 10.1038/nature11582 PMC3491803

[DAVISONGR220111C35] Kamada N, Seo SU, Chen GY, Nunez G. 2013 Role of the gut microbiota in immunity and inflammatory disease. Nat Rev Immunol 13: 321–335. 23618829 10.1038/nri3430

[DAVISONGR220111C36] Kanther M, Sun X, Muhlbauer M, Mackey LC, Flynn EJ III, Bagnat M, Jobin C, Rawls JF. 2011 Microbial colonization induces dynamic temporal and spatial patterns of NF-κB activation in the zebrafish digestive tract. Gastroenterology 141: 197–207. 21439961 10.1053/j.gastro.2011.03.042 PMC3164861

[DAVISONGR220111C38] Korecka A, de Wouters T, Cultrone A, Lapaque N, Pettersson S, Dore J, Blottiere HM, Arulampalam V. 2013 ANGPTL4 expression induced by butyrate and rosiglitazone in human intestinal epithelial cells utilizes independent pathways. Am J Physiol Gastrointest Liver Physiol 304: G1025–G1037. 23518684 10.1152/ajpgi.00293.2012

[DAVISONGR220111C39] Krautkramer KA, Kreznar JH, Romano KA, Vivas EI, Barrett-Wilt GA, Rabaglia ME, Keller MP, Attie AD, Rey FE, Denu JM. 2016 Diet-microbiota interactions mediate global epigenetic programming in multiple host tissues. Mol Cell 64: 982–992. 27889451 10.1016/j.molcel.2016.10.025 PMC5227652

[DAVISONGR220111C40] Li Y, Rao X, Mattox WW, Amos CI, Liu B. 2015 RNA-seq analysis of differential splice junction usage and intron retentions by DEXSeq. PLoS One 10: e0136653. 26327458 10.1371/journal.pone.0136653 PMC4556662

[DAVISONGR220111C41] Love MI, Huber W, Anders S. 2014 Moderated estimation of fold change and dispersion for RNA-seq data with DESeq2. Genome Biol 15: 550. 25516281 10.1186/s13059-014-0550-8 PMC4302049

[DAVISONGR220111C42] Ma R, Yang H, Li J, Yang X, Chen X, Hu Y, Wang Z, Xue L, Zhou W. 2016 Association of HNF4α gene polymorphisms with susceptibility to type 2 diabetes. Mol Med Rep 13: 2241–2246. 26781905 10.3892/mmr.2016.4780

[DAVISONGR220111C43] Marcil V, Seidman E, Sinnett D, Boudreau F, Gendron FP, Beaulieu JF, Menard D, Precourt LP, Amre D, Levy E. 2010 Modification in oxidative stress, inflammation, and lipoprotein assembly in response to hepatocyte nuclear factor 4α knockdown in intestinal epithelial cells. J Biol Chem 285: 40448–40460. 20871093 10.1074/jbc.M110.155358 PMC3003343

[DAVISONGR220111C44] Marcil V, Sinnett D, Seidman E, Boudreau F, Gendron FP, Beaulieu JF, Menard D, Lambert M, Bitton A, Sanchez R, 2012 Association between genetic variants in the HNF4A gene and childhood-onset Crohn's disease. Genes Immun 13: 556–565. 22914433 10.1038/gene.2012.37 PMC4931920

[DAVISONGR220111C45] Marjoram L, Alvers A, Deerhake ME, Bagwell J, Mankiewicz J, Cocchiaro JL, Beerman RW, Willer J, Sumigray KD, Katsanis N, 2015 Epigenetic control of intestinal barrier function and inflammation in zebrafish. Proc Natl Acad Sci 112: 2770–2775. 25730872 10.1073/pnas.1424089112 PMC4352795

[DAVISONGR220111C46] Mbodji K, Charpentier C, Guerin C, Querec C, Bole-Feysot C, Aziz M, Savoye G, Dechelotte P, Marion-Letellier R. 2013 Adjunct therapy of n-3 fatty acids to 5-ASA ameliorates inflammatory score and decreases NF-κB in rats with TNBS-induced colitis. J Nutr Biochem 24: 700–705. 22841543 10.1016/j.jnutbio.2012.03.022

[DAVISONGR220111C47] Meddens CA, Harakalova M, van den Dungen NA, Foroughi Asl H, Hijma HJ, Cuppen EP, Bjorkegren JL, Asselbergs FW, Nieuwenhuis EE, Mokry M. 2016 Systematic analysis of chromatin interactions at disease associated loci links novel candidate genes to inflammatory bowel disease. Genome Biol 17: 247. 27903283 10.1186/s13059-016-1100-3 PMC5131449

[DAVISONGR220111C48] Mokry M, Middendorp S, Wiegerinck CL, Witte M, Teunissen H, Meddens CA, Cuppen E, Clevers H, Nieuwenhuis EE. 2014 Many inflammatory bowel disease risk loci include regions that regulate gene expression in immune cells and the intestinal epithelium. Gastroenterology 146: 1040–1047. 24333384 10.1053/j.gastro.2013.12.003

[DAVISONGR220111C49] Morgun A, Dzutsev A, Dong X, Greer RL, Sexton DJ, Ravel J, Schuster M, Hsiao W, Matzinger P, Shulzhenko N. 2015 Uncovering effects of antibiotics on the host and microbiota using transkingdom gene networks. Gut 64: 1732–1743. 25614621 10.1136/gutjnl-2014-308820 PMC5166700

[DAVISONGR220111C50] Nijmeijer RM, Gadaleta RM, van Mil SW, van Bodegraven AA, Crusius JB, Dijkstra G, Hommes DW, de Jong DJ, Stokkers PC, Verspaget HW, 2011 Farnesoid X receptor (FXR) activation and FXR genetic variation in inflammatory bowel disease. PLoS One 6: e23745. 21887309 10.1371/journal.pone.0023745 PMC3161760

[DAVISONGR220111C51] O'Shea EF, Cotter PD, Stanton C, Ross RP, Hill C. 2012 Production of bioactive substances by intestinal bacteria as a basis for explaining probiotic mechanisms: bacteriocins and conjugated linoleic acid. Int J Food Microbiol 152: 189–205. 21742394 10.1016/j.ijfoodmicro.2011.05.025

[DAVISONGR220111C52] Palanker L, Tennessen JM, Lam G, Thummel CS. 2009 *Drosophila* HNF4 regulates lipid mobilization and β-oxidation. Cell Metab 9: 228–239. 19254568 10.1016/j.cmet.2009.01.009 PMC2673486

[DAVISONGR220111C53] Plevy S, Silverberg MS, Lockton S, Stockfisch T, Croner L, Stachelski J, Brown M, Triggs C, Chuang E, Princen F, 2013 Combined serological, genetic, and inflammatory markers differentiate non-IBD, Crohn's disease, and ulcerative colitis patients. Inflamm Bowel Dis 19: 1139–1148. 23518807 10.1097/MIB.0b013e318280b19e PMC3792797

[DAVISONGR220111C54] Qin J, Li Y, Cai Z, Li S, Zhu J, Zhang F, Liang S, Zhang W, Guan Y, Shen D, 2012 A metagenome-wide association study of gut microbiota in type 2 diabetes. Nature 490: 55–60. 23023125 10.1038/nature11450

[DAVISONGR220111C55] Rabot S, Membrez M, Bruneau A, Gerard P, Harach T, Moser M, Raymond F, Mansourian R, Chou CJ. 2010 Germ-free C57BL/6J mice are resistant to high-fat-diet-induced insulin resistance and have altered cholesterol metabolism. FASEB J 24: 4948–4959. 20724524 10.1096/fj.10-164921

[DAVISONGR220111C56] Rawls JF, Samuel BS, Gordon JI. 2004 Gnotobiotic zebrafish reveal evolutionarily conserved responses to the gut microbiota. Proc Natl Acad Sci 101: 4596–4601. 15070763 10.1073/pnas.0400706101 PMC384792

[DAVISONGR220111C57] Rha GB, Wu G, Shoelson SE, Chi YI. 2009 Multiple binding modes between HNF4α and the LXXLL motifs of PGC-1α lead to full activation. J Biol Chem 284: 35165–35176. 19846556 10.1074/jbc.M109.052506 PMC2787377

[DAVISONGR220111C58] San Roman AK, Aronson BE, Krasinski SD, Shivdasani RA, Verzi MP. 2015 Transcription factors GATA4 and HNF4A control distinct aspects of intestinal homeostasis in conjunction with transcription factor CDX2. J Biol Chem 290: 1850–1860. 25488664 10.1074/jbc.M114.620211 PMC4340426

[DAVISONGR220111C59] Sartor RB, Wu GD. 2016 Roles for intestinal bacteria, viruses, and fungi in pathogenesis of inflammatory bowel diseases and therapeutic approaches. Gastroenterology 152: 327–339.e4. 27769810 10.1053/j.gastro.2016.10.012 PMC5511756

[DAVISONGR220111C60] Semenkovich NP, Planer JD, Ahern PP, Griffin NW, Lin CY, Gordon JI. 2016 Impact of the gut microbiota on enhancer accessibility in gut intraepithelial lymphocytes. Proc Natl Acad Sci 113: 14805–14810. 27911843 10.1073/pnas.1617793113 PMC5187723

[DAVISONGR220111C61] Semova I, Carten JD, Stombaugh J, Mackey LC, Knight R, Farber SA, Rawls JF. 2012 Microbiota regulate intestinal absorption and metabolism of fatty acids in the zebrafish. Cell Host Microbe 12: 277–288. 22980325 10.1016/j.chom.2012.08.003 PMC3517662

[DAVISONGR220111C62] Shulzhenko N, Morgun A, Hsiao W, Battle M, Yao M, Gavrilova O, Orandle M, Mayer L, Macpherson AJ, McCoy KD, 2011 Crosstalk between B lymphocytes, microbiota and the intestinal epithelium governs immunity versus metabolism in the gut. Nat Med 17: 1585–1593. 22101768 10.1038/nm.2505 PMC3902046

[DAVISONGR220111C63] Soutoglou E, Katrakili N, Talianidis I. 2000 Acetylation regulates transcription factor activity at multiple levels. Mol Cell 5: 745–751. 10882110 10.1016/s1097-2765(00)80253-1

[DAVISONGR220111C64] Staiger H, Haas C, Machann J, Werner R, Weisser M, Schick F, Machicao F, Stefan N, Fritsche A, Haring HU. 2009 Muscle-derived angiopoietin-like protein 4 is induced by fatty acids via peroxisome proliferator-activated receptor (PPAR)-δ and is of metabolic relevance in humans. Diabetes 58: 579–589. 19074989 10.2337/db07-1438 PMC2646056

[DAVISONGR220111C65] Stegmann A, Hansen M, Wang Y, Larsen JB, Lund LR, Ritie L, Nicholson JK, Quistorff B, Simon-Assmann P, Troelsen JT, 2006 Metabolome, transcriptome, and bioinformatic *cis*-element analyses point to HNF-4 as a central regulator of gene expression during enterocyte differentiation. Physiol Genomics 27: 141–155. 16868071 10.1152/physiolgenomics.00314.2005

[DAVISONGR220111C66] Thaiss Christoph A, Levy M, Korem T, Dohnalová L, Shapiro H, Jaitin Diego A, David E, Winter Deborah R, Gury-BenAri M, Tatirovsky E, 2016 Microbiota diurnal rhythmicity programs host transcriptome oscillations. Cell 167: 1495–1510.e12. 27912059 10.1016/j.cell.2016.11.003

[DAVISONGR220111C67] Trapnell C, Hendrickson DG, Sauvageau M, Goff L, Rinn JL, Pachter L. 2013 Differential analysis of gene regulation at transcript resolution with RNA-seq. Nat Biotech 31: 46–53. 10.1038/nbt.2450 PMC3869392 23222703

[DAVISONGR220111C68] Tremblay E, Thibault MP, Ferretti E, Babakissa C, Bertelle V, Bettolli M, Burghardt KM, Colombani JF, Grynspan D, Levy E, 2016 Gene expression profiling in necrotizing enterocolitis reveals pathways common to those reported in Crohn's disease. BMC Med Genomics 9: 6. 26801768 10.1186/s12920-016-0166-9 PMC4722613

[DAVISONGR220111C69] Veilleux A, Mayeur S, Berube JC, Beaulieu JF, Tremblay E, Hould FS, Bosse Y, Richard D, Levy E. 2015 Altered intestinal functions and increased local inflammation in insulin-resistant obese subjects: a gene-expression profile analysis. BMC Gastroenterol 15: 119. 26376914 10.1186/s12876-015-0342-y PMC4574092

[DAVISONGR220111C70] Vrieze A, Van Nood E, Holleman F, Salojarvi J, Kootte RS, Bartelsman JF, Dallinga-Thie GM, Ackermans MT, Serlie MJ, Oozeer R, 2012 Transfer of intestinal microbiota from lean donors increases insulin sensitivity in individuals with metabolic syndrome. Gastroenterology 143: 913–916.e7. 22728514 10.1053/j.gastro.2012.06.031

[DAVISONGR220111C71] Weissglas-Volkov D, Huertas-Vazquez A, Suviolahti E, Lee J, Plaisier C, Canizales-Quinteros S, Tusie-Luna T, Aguilar-Salinas C, Taskinen MR, Pajukanta P. 2006 Common hepatic nuclear factor-4α variants are associated with high serum lipid levels and the metabolic syndrome. Diabetes 55: 1970–1977. 16804065 10.2337/db06-0035

[DAVISONGR220111C72] Yuan X, Ta TC, Lin M, Evans JR, Dong Y, Bolotin E, Sherman MA, Forman BM, Sladek FM. 2009 Identification of an endogenous ligand bound to a native orphan nuclear receptor. PLoS One 4: e5609. 19440305 10.1371/journal.pone.0005609 PMC2680617

[DAVISONGR220111C73] Zhang Y, Liu T, Meyer CA, Eeckhoute J, Johnson DS, Bernstein BE, Nusbaum C, Myers RM, Brown M, Li W, 2008 Model-based analysis of ChIP-Seq (MACS). Genome Biol 9: R137. 18798982 10.1186/gb-2008-9-9-r137 PMC2592715

