# Supplementary material for: Microbiota regulate intestinal epithelial gene expression by suppressing the transcription factor Hepatocyte nuclear factor 4 alpha
Source: Genome Res. 2017 Jul;27(7):1195–206. doi: 10.1101/gr.220111.116 (PMC5495071; doi:10.1101/gr.220111.116)
Supplement: Supplemental Material [file supp_gr.220111.116_Supplemental_Material.pdf]

SUPPLEMENTAL MATERIAL

Microbiota regulate intestinal epithelial gene expression by suppressing the transcription factor Hepatocyte nuclear factor 4 alpha

James M. Davison, Colin R. Lickwar, Lingyun Song, Ghislain Breton, Gregory E. Crawford and John F. Rawls

Supplemental Figure Summary

FIGURE S1: The *hnf4* family of transcription factors bind specifically to a microbiota suppressed zebrafish enhancer.

FIGURE S2: *hnf4a*$^{-43/-43}$ mutants survive to adulthood and have reduced *hnf4a* transcript and reduced intestinal lumen size.

FIGURE S3: Hnf4a maintains transcriptional homeostasis in the presence of a microbiota in zebrafish digestive tracts.

FIGURE S4: HNF4A and STAT binding sites are enriched with within promoters of microbiota suppressed and induced genes, respectively.

FIGURE S5: HNF4 GF ChIP-Seq replicates have reproducibly higher signal than HNF4 CV ChIP-seq replicates.

FIGURE S6: Microbiota suppress HNF4A and HNF4G activity without overtly impacting protein levels or localization.

FIGURE S7: Model of microbiota regulation of host gene transcription through modification of enhancer activity and suppression of HNF4A DNA binding.

Supplemental Table Summary with Description

Table S1: Transcription factors in the ORFEOME Y1H library

- *This table contains the list of all of the zebrafish transcription factors that were in the Y1H prey vector library.*

Table S2: Cuffdiff results from gnotobiotic WT and mutant zebrafish RNA-seq

- *This table contains the results from the Cuffdiff analysis in the gnotobiotic WT and mutant zebrafish digestive tracts.*

Table S3: Cuffdiff results from gnotobiotic mouse jejunal IEC RNA-seq

- *This table contains the results from the Cuffdiff analysis from jejunal IECs from CV and GF mice*

Table S4: Summary of from differential zebrafish gene lists

- *This multi-tab spreadsheet contains Ensemble Gene IDs from the differential gene lists (i-vi) located within Supplemental Fig. S3D.*

Table S5: Total number of MACS2 peak calls per ChIP per replicate

- *Number of peaks indicated are raw results generated from MACS2 and peaks generated by background signal/noise have not been filtered out. All peaks generated by background sequencing noise were removed manually for downstream analysis*

Table S6: Genes associated with differential DNase and Histone Marks

- *This multi-tab spreadsheet contains the genes associated with differential DHSs (as determined by DESeq2, p-value < 0.01), H3K4me1 and H3k27ac sites (as determined by DESeq2 FDR < 0.01). Gene associations were determined by GREAT version 3.0.0 using a 10,000 bp nearest gene rule.*

Table S7: Genes associated with GF and CV HNF4A sites

- *This multi-tab spreadsheet contains the genes associated with HNF4A sites in GF jejunal IECs and CV jejunal IECs. Gene associations were determined by GREAT version 3.0.0 using a 10,000 bp nearest gene rule.*

Table S8: Genomic coordinates of differential DNase, histone marks and the location of GF and CV *HNF4A* sites

- *This multi-tab spreadsheet contains the genomic coordinates of differential DHSs (as determined by DESeq2, p-value < 0.01), H3K4me1 and H3K27ac sites (as determined by DESeq2 FDR < 0.01). Also included are the genomic coordinates for all HNF4A sites used in Fig. 4 and Supplemental Fig. S6.*

Table S9: Primers and oligos

- *This spreadsheet contains the sequences of the primers and oligonucleotides used in the Y1H, site directed mutagenesis, zebrafish mutagenesis, ChIP-PCR and qRT-PCR.*

Table S10: Protein sequences used for the phylogeny tree

- *This spreadsheet contains HNF4 protein sequences used to generate the phylogeny tree in Supplemental Fig. S1G.*

Table S11: Diseases related to 88 genes that are suppressed by zebrafish microbiota but activated by *hnf4a*.

- *This table contains the diseases related to the 88 genes that are suppressed by the zebrafish microbiota and activated by hnf4a.*

SUPPLEMENTAL METHODS:

Zebrafish Husbandry:

Tg(in3.4:cfos:gfp) (Camp et al. 2012) stable transgenic lines were maintained on a TL/Tü background using established protocols approved by the Animal Studies Committee at the University of North Carolina at Chapel Hill and Duke University School of Medicine. Conventionally raised zebrafish were reared and maintained as described (Westerfield 2000). Production, colonization, maintenance, and sterility testing of gnotobiotic zebrafish were performed as described (Pham et al. 2008).

Mouse Husbandry:

All mouse husbandry was performed as described in (Camp et al. 2014) using established protocols approved by the Animal Studies Committee at the University of North Carolina at Chapel Hill and Duke University School of Medicine with the following exceptions. All mice used in this study were 10 – 12 week old male C57BL/6J, housed on Alpha-dri bedding (Shepherd) and fed 2020SX diet (Envigo) *ad libitum*. To generate conventionalized mice, germ-free mice were colonized with a conventional microbiota from by receiving a 200 µL oral gavage of 20% glycerol stock containing 1:1 w/v fecal sample collected from adult SPF C57BL/6J mice collected over 2 weeks and homogenized in reduced PBS.

Yeast 1-Hybrid ORFeome Screen:

The yeast 1-hybrid ORFeome screen was performed using the Clontech MatchmakerTM Gold Yeast One-hybrid Library Screening System (cat. 630491) protocol with the following exceptions: The Y1HGold yeast strain was transformed using standard yeast transformation procedures with BstBI digested pBait-AbAi containing either the WT or a SDM in3.4 or the p53 binding site sequence, and positive transformants were selected on SD/-URA media. In

addition, a ORFeome library consisting of 148 zebrafish transcription factors cloned from adult zebrafish liver (Supplemental Table S1) into pDEST22 prey vectors containing an N-terminal GAL4-activation domain was utilized (Boyle et al. 2017). Each plasmid was individually transformed in the yeast strains Y1HGold[in3.4/AbAi] or Y1HGold[p53/AbAi] and positive transformants were selected on SD -URA -TRP. The primary screen to test for positive interactions between the prey transcription factor and the bait sequence was tested twice in the laboratory by pipetting 10 µL of transformed yeast onto SD/-URA/-TRP with AbA (125ng/ml) agarose plates. The secondary screen to test for positive interactions was performed by streaking individual colonies from the primary screen onto SD/-URA/-TRP with AbA (125ng/ml) agarose plates. Zebrafish *hnf4a* and *hnf4g* cDNAs (see Supplemental Table S9 for primers used for amplification) were cloned into a custom pENTR plasmid (termed pENTR-Ale1) using In Fusion (Takara Bio 638909) and inserted into pDEST22 using LR clonase (Invitrogen 12538120). These newly cloned transcription factors were tested for a positive interaction with in3.4 using the same procedures as above.

Site Directed Mutagenesis:

Site directed mutagenesis was performed using the primers found in Supplemental Table S9. A 40 cycle PCR reaction was performed using iProof HiFi Polymerase (Biorad 1725301). Newly synthesized plasmids were digested with DpnI (New England Biolabs R0176L) overnight to digest template DNA and transformed into DH5a *E. coli*. SDM Vectors were confirmed by Sanger sequencing.

Zebrafish Transgenesis and Imaging:

Co-injections of Tol2 SDM or WT in3.4:cfos:gfp plasmid and transposase mRNA were performed as described (Camp et al. 2012) with the following exceptions: 50 – 100 zebrafish embryos were injected at the 1–2 cell stage with approximately 69 pg of plasmid DNA at a

DNA:transposase ratio of 1:2. At least 9 - 18 fish/construct were imaged on a Leica M205 FA with a Leica DFC 365FX camera at the same magnification and exposure time and densitometric measures were quantified in 8-bit gray scale images using FIJI software. Three mosaic patches within a given tissue of an imaged fish were quantified for mean fluorescence intensity and averaged. Statistical significance was analyzed using Kruskal-Wallis one-way analysis of variance and Dunn's multiple comparison test using GraphPad Prism software.

Zebrafish Mutagenesis:

Targeted gene deletion of the *hnf4a* gene was performed using CRISPR/Cas9 nuclease RNA-guided genome editing targeting the fourth exon of *hnf4a*. The guide RNA sequences were designed using "CRISPR Design Tool" (http://crispr.mit.edu/). Guide RNAs (Supplemental Table S9) were generated from BamHI (New England Biolabs R0136L) digested pT7-gRNA plasmid (a gift from Wenbiao Chen and available from Addgene: http://www.addgene.org/46759/) and by performing an *in vitro* transcription reaction using MEGAshortscript T7 kit (Ambion/Invitrogen AM1354) (Jao et al. 2013). Cas9 mRNA was generated from XbaI (New England Biolabs R0145S) digested pT3TS-nls-zCas9-nls plasmid (a gift from Wenbiao Chen and available from Addgene: http://www.addgene.org/46757/) followed by an *in vitro* transcription reaction using mMESSAGE mMACHINE T3 kit (Ambion/Invitrogen AM1348) (Jao et al. 2013). 150 ng/µL of nls-zCas9-nls and 34 ng/µL of each gRNA, 0.05% phenol red, 120 mM KCl, and 20mM Hepes (pH 7.0) were injected directly into the cell(s) of one to two-cell stage zebrafish embryos of Tü background. Mutagenesis was initially screened using Melt Doctor High Resolution Melting Assay (Thermo Fisher Scientific 4409535) and subsequent screening of the -43 (allele designation *rdu14*) and +25 (allele designation *rdu15*) alleles was performed using 2% agarose sodium borate gel electrophoresis. Protein and DNA sequences were visualized using CLC Sequence Viewer 7 (CLC Bio). The HNF4 protein phylogram was generated in CLC Sequence Viewer 7 using sequences listed in Supplemental

Table S10. Sequences were aligned using default settings and the tree was constructed using the UPGMA method and Jukes-Cantor settings for protein distance measurements. The majority of zebrafish experiments were performed using *hnf4a*$^{-43/-43}$ genotype. However, to increase sample sizes, gnotobiotic zebrafish experiments were performed using larvae with *hnf4a*$^{-43/-43}$ and *hnf4a*$^{-43/+25}$ genotypes. We have not observed significant differences in gene expression or morphology between *hnf4a*$^{-43/-43}$ and *hnf4a*$^{+25/+25}$ genotypes.

Zebrafish Immunohistochemistry:

Zebrafish larvae at 6 dpf were fixed in 4% PFA overnight at 4 C. Fixed larvae were mounted in 4% low melting point agarose molds. 200 µm axial cross sections of fixed larvae were generated using a Leica VT1000S. Vibratome slices were washed once in ice cold PBS followed by 4 times with PBS containing 0.1% tween 20 and then incubated in blocking solution (PBS with 10% heat inactivated calf serum, 0.1% Tween-20 and 0.5% Triton X-100) for 4 hours. Slices were incubated overnight with 4e8 antibody (Mouse anti-4e8, Abcam ab73643) diluted 1:200 in PBS with 5% heat inactivated calf serum, 0.1% Tween-20 and 0.5% Triton X-100 at 4°C with agitation. Samples were washed in PBST 3 times for 10 minutes per wash and incubated with secondary antibody (1:1000) (Goat Anti-Mouse Alexa Fluor 568 Invitrogen, A11004) and Alexa Fluor 647 phalloidin (1:300) (Invitrogen, A22287) in PBS with 5% heat inactivated calf serum, 0.1% Tween-20 and 0.5% Triton X-100 for 3 hours. Slices were washed in PBS 3 times for ten minutes per wash, mounted onto slides with DAPI mounting media (Vector Laboratories, Inc, H-1200) and imaged on a Leica SP8 confocal microscope. Images shown in Fig. 1F are representative of two experiments with 3 larvae per experiment per genotype.

Mouse IEC Isolation:

Mice were euthanized under CO_2 and cervical dislocation and placed on a chilled wax dissection pad. The small intestine was removed from the mouse and the jejunum was excised from the duodenum and ileum. Duodenum was defined as the anterior 5 cm of the midgut and ileum was defined as posterior 6 cm of midgut as described (Camp et al. 2014). Adipose and vasculature were removed from the tissue. The jejunum was opened longitudinally along the length of the tissue, exposing the lumen and epithelial cell layer. Luminal debris was washed away from the epithelia with ice cold sterile PBS. The tissue was temporarily stored in 10 ml of ice cold sterile PBS with 1x Protease Inhibitors (cOmplete EDTA-Free Protease Inhibitor Cocktail, Roche 11873580001) and 10 µM Y-27632 (ROCK I inhibitor, Selleck Chemicals S1049) to inhibit spontaneous apoptosis. The jejunum was moved into a 15 ml conical tube containing 3 mM EDTA in PBS with 1x Protease Inhibitors and 10 µM Y-27632. The tissue was placed on a nutator in a cold room for 15 minutes. The jejunum was removed from the 3 mM EDTA and placed on an ice cold glass petri dish with PBS containing 1 mM $MgCl_2$ and 2 mM $CaCl_2$ with 1X Protease Inhibitors and 10 µM Y-27632. Villi were scraped off of the tissue using the side of a sterile plastic micropipette and transferred into a new 15 ml conical tube. The isolated IECs were then pelleted at 250 x g at 4°C for 5 minutes, resuspended in 15 ml of ice cold PBS containing 10 µM Y-27632 and 1x Protease Inhibitors and pelleted again at 250 x *g* at 4°C. The cell pellet was used for chromatin immunoprecipitation or for nuclear extractions.

Mouse Intestine Immunofluorescence and Western Blot:

Mid-jejunal tissue was dissected and cleaned as in the IEC villi isolation above. The whole, splayed open tissue was pinned to 3% agarose and fixed in 4% PFA overnight with gentle agitation at 4°C. The fixed tissue was washed 4 times with PBS for 15 minutes. The tissue was then permeabilized in PBS with 0.5% Tween 20 for 1.5 hours at room temperature. Following permeabilization, the tissue was blocked in 5% donkey serum in PBS with 0.1% Tween 20 for 2

hours at room temperature. The tissue was moved into a 35 mm dish and incubated with the primary antibody (Mouse anti-HNF4A, Abcam 41898 or Goat anti-HNF4G, Santa Cruz sc-6558, Lot I299) diluted 1:200 overnight at 4°C with gentle agitation. The tissue was washed 4 times in immunowash buffer (PBS, 0.1% Tween-20, 1% DMSO and 1% BSA) at room temperature and incubated in secondary antibody (Goat Anti-Mouse Alexa Fluor 488 Invitrogen, A11001 or Donkey Anti-Goat Alex Fluor 568 Invitrogen, A11057) diluted 1:100 and Alexa Fluor 488 phalloidin or Alexa Fluor 568 phalloidin diluted 1:250 (Invitrogen A12379 and A12380, respectively) in PBS with 0.1% Tween-20 for 4.5 hours at room temperature. The tissue was then washed 6x in immunowash buffer, mounted on a microscope slide with DAPI mounting media (Vector Laboratories Inc., H-1200) and imaged on a Leica SP8 confocal microscope.

Western blots were performed on non-crosslinked IEC lysates (see below) using standard chemoluminescence Western blot protocols, including ECL (Biorad 170-5061) and primary antibodies Goat anti-HNF4A (Santa Cruz sc-6556, Lot A2215), Goat anti-HNF4G (Santa Cruz sc-6558, Lot I299) and Rabbit anti-ACTB (Cell Signaling 13E5), Donkey anti-Goat-HRP conjugate (Santa Cruz sc-2020), and Goat anti-Rabbit-HRP conjugate (Jackson ImmunoResearch 111-035-003, a gift from Stacy Horner at Duke University). The western blot shown in Supplemental Fig. S6H is representative of two experiments.

Cell Lysis and Chromatin Sonication for ChIP:

Washed and pelleted IECs were resuspended in 10 ml of 1% EM grade formaldehyde (Electron Microscopy sciences, 15710) in ice cold PBS containing 10 µM Y-27632 and 1x Protease Inhibitors. The cells were fixed for 25 minutes at room temperature with agitation. Formaldehyde fixation was quenched by adding glycine to a final concentration of 125 mM. The cells were pelleted at 250 x g for 5 minutes at 4°C and resuspended in ice cold PBS with 1x Protease Inhibitors and 10 µM Y-27632. This wash step was repeated twice. Upon the third wash, the cell pellet was aliquoted into 3 equal volumes in 3 microfuge tubes. The cells were

pelleted in the microfuge tubes and resuspended in 300 µL of ChIP Lysis Buffer (1% IGEPAL, 0.5% sodium deoxycholate, 1% SDS in 1x PBS) containing 1x Protease Inhibitors. The cells were stored on ice for 2 hours and sonicated using a Diagenode Bioruptor 4°C water bath sonicator. Chromatin was sheared to mean size of 250 – 300 bp (10 minutes of 30 seconds on High, 30 seconds off, repeated once for a total of 20 minutes – total sonication time is 10 minutes on High, 10 minutes off). Sonicated material was spun at 14,000 x *g* for 20 minutes and the supernatant was transferred to a new microfuge tube. ChIP was performed immediately on sonicated chromatin or it was snap frozen and stored at -80°C. To check chromatin shearing efficiency and to prepare ChIP input samples, 20 µL of each sonicated sample was removed and added to a new tube. 180 µL of ChIP elution buffer and 8 µL of 5 M NaCl was added to the 20 µL input samples. Chromatin shearing efficiency was visualized on a gel following reverse crosslinking by incubating the input sample at 65°C overnight**.**

Chromatin Immunoprecipitation, Library Preparation and Next-Generation Sequencing:

Frozen sonicated chromatin was thawed on ice. Thawed and fresh chromatin samples were diluted in 1 mL of ChIP dilution buffer (1% Triton X-100, 2 mM EDTA, 20 mM Tris-Cl (pH 8.1), and 150 mM NaCl) containing 1x Protease Inhibitor and precleared with washed protein G Dynabeads (Thermo Fisher Scientific 10004D) for 3 hours at 4°C on a nutator. Precleared chromatin was incubated with ChIP grade antibodies [4 µg H3K4me1 (Rabbit anti-H3K4me1, Abcam ab8895), 4 µg H3K27ac (Rabbit anti-H3K27ac, Abcam ab4729), 8 µg HNF4A (Mouse anti-HNF4A, Abcam 41898), 8 µg HNF4G (Goat anti-HNF4G, Santa Cruz sc-6558X), 8 µg CTCF (Rabbit anti-CTCF, Active Motif, 61311, Lot 34614003)] overnight at 4°C on a nutator. Antibody-chromatin complexes were pulled down with washed protein G dynabeads for 4 hours at 4°C on a nutator. The beads were washed 5x for 3 minutes with ice cold LiCl wash buffer (100 mM Tris-Cl (pH 7.5), 500 mM LiCl, 1% IGEPAL, 1% sodium deoxycholate) and 1x with ice cold TE buffer at 4°C on a nutator. Washed beads were resuspended in 100 µL of ChIP elution

buffer (1% SDS and 0.1 M sodium bicarbonate) and placed in a Eppendorf ThermoMixer C heated to 65°C and programed to vortex at 2000 RPM for 15 seconds, rest for 2 minutes for a total of 30 minutes. The beads were pelleted, placed on a magnet, and the supernatant was moved to a new tube. This elution process was repeated once and corresponding elutions were combined for a total of 200 µL. To reverse crosslink immunoprecipitated chromatin, 8 µL of 5 M NaCl was added to each 200 µL ChIP elution and elutions were incubated at 65°C overnight. Immunoprecipitated chromatin was isolated using a QIAquick PCR quick preparation kit (Qiagen 28104), quantified using a Qubit 2.0 flourometer and stored at -80°C until library preparations and amplification. Libraries were always prepared within 3 days of the immunoprecipitation with the NEBNextUltra DNA Library Prep Kit for Illumina (New England Biolabs E7370S). Prepared libraries were quantified using a Qubit 2.0 fluorometer and submitted to Hudson Alpha Genomic Services Laboratory for 50 bp single end sequencing on an Illumina HiSeq 2500 with 4 samples per lane in the flow cell.

Germ-free or conventionalized chromatin for input normalization was generated using the same protocol as above except no antibody was used during the overnight antibody incubation; instead, chromatin was incubated at 4°C with gentle agitation. Bead incubation, reverse-crosslinking and library preparations for these samples were performed using the same protocol as the ChIPs.

DNase Hypersensitivity on IECs:

DNase hypersensitivity was performed as described (Camp et al. 2014) with the following modifications: IECs were isolated as above from jejunum and subjected to endogenous DNase activity to digest chromatin. DNase-seq libraries were constructed as previously described, with "Oligo 1b" phosphorylated at the 5'-end to enhance ligation efficiency (Song and Crawford 2010). Libraries were sequenced by Illumina HiSeq 2000 with 50 bp single end reads with 3 samples per lane at the Duke Sequencing and Genomic Technologies Core.

RNA Isolation, qRT-PCR, RNA-seq:

For RNA-seq, zebrafish digestive tracts (including whole intestine, liver, pancreas, gall bladder; n = 13 – 20 per condition per genotype) were removed by microdissection and resuspended in 1 mL TRIzol (Ambion/Invitrogen/ThermoFisher Scientific 15596026). Larval digestive tracts were lysed by being passing through a 25 G needle followed by a 27.5 G needle 5 times each. Mouse jejunum intestinal epithelial cells were collected as described above. Prior to crosslinking, 1/50 of the isolated IECs were suspended in 1 ml TRIzol and stored at -80°C. For both zebrafish and mouse RNA samples, 200 µL of chloroform was added to the TRIzol and the sample was vortexed on high for 30 seconds at room temperature. The samples were incubated at room temperature for 2 minutes and centrifuged at 12,000 x *g* for 15 minutes at 4°C. The top aqueous layer was removed and added to equal volume of isopropanol. The nucleic acids were isolated using a column-based RNA-isolation kit (Ambion Cat 12183018A) with an on-column DNase I (RNase-free) treatment (New England Biolabs M0303L) to remove DNA contamination. RNA was eluted off the column in nuclease-free water, quantified using a Qubit 2.0 and stored at -80°C until submission to the Duke Sequencing and Genomic Technologies Core. RNA-seq libraries were prepared and sequenced by Duke Sequencing and Genomic Technologies Core on an Illumina HiSeq 2500 with 4 samples per lane in the flow cell.

For qRT-PCR, adult zebrafish IECs from one adult or 6 dpf larval digestive tracts (n= 5 – 10) with the same genotype were suspended in 1 mL of TRIzol. RNA was extracted using the same protocol as above with the following exceptions: following sample resuspension in isopropanol, samples were frozen at -20°C O/N and spun at 15,000 x *g* for 30 minutes. Pellets were washed twice with RNase-free 70% ethanol and left to air dry for 10 minutes. Nucleic acids were resuspended in RNase-free water containing DNase (DNA-free DNA Removal Kit, ThermoFisher Scientific AM1906) and incubated for 30 minutes at 37°C. DNase was then inhibited per manufacturer instructions and purified RNA was quantified using a Nanodrop

spectrophotometer and stored at -80°C. cDNA was generated using iScript cDNA synthesis kit (Biorad 1708891) and qRT-PCR was performed using Quanta's PerfeCTa Sybr-green (Quanta 101414-154) in an Applied Biosystems StepOnePlus Real-Time PCR Systems machine (Supplemental Table S9).

For ChIP-PCR, immunoprecipitated chromatin was isolated using a QIAquick PCR quick preparation kit (Qiagen 28104), and stored at -20°C. Immunoprecipitated chromatin was used as template in a qRT-PCR reaction using Quanta's PerfeCTa Sybr-green (Quanta 101414-154) in an Applied Biosystems StepOnePlus Real-Time PCR Systems machine (Supplemental Table S9).

RNA-seq Bioinformatics:

Zebrafish RNA-seq reads were aligned to the zebrafish genome (danRer7) using TopHat2 v0.6 using *de novo* splice junction mapping (default TopHat settings). FPKM expression values were obtained for transcripts via Cufflinks, and pairwise differential gene expression tests were carried out with Cuffdiff v0.0.6 (Trapnell et al. 2012) using a minimum alignment count of 100 and using multi-read correct and read group datasets. The default significance threshold of FDR < 5% was used for each comparison. Hierarchical clustering of replicates and gene expression heat map of RNA-seq data were generated using complete linkage clustering and averaging the $\log_{10}$ (FPKM) of each gene with Cluster v3.0. Subsequent heat maps in Supplemental Fig. S3 were generated using complete hierarchical gene clustering of the $\log_2$ (fold change) between compared conditions with Cluster v3.0. Principle components analysis was performed with a wide estimation method with JMP13. Discriminant analysis was performed with a wide linear method with JMP13. GO enrichments were performed with DAVID 6.7 (Huang da et al. 2009a; Huang da et al. 2009b). The relatedness heat map was generated with principle components in R (ggplot2 package). A 4-way Venn diagram was generated using the 4,007 genes that were differentially regulated in any comparisons with online software:

http://bioinformatics.psb.ugent.be/webtools/Venn/. Disease associations were performed using DRSC Disease Gene Query Tool (Hu et al. 2011) (http://www.flyrnai.org/diopt-dist). The number of genes associated with various diseases were added together and ranked by total number of associated genes. Unique genes associated with “Inflammatory Bowel Disease”, “Crohn’s Disease”, and “Ulcerative Colitis” were combined into one IBD list. If a zebrafish gene was orthologous to many human gene associated with the same gene, the zebrafish gene was counted only once for a given disease. For example, *fads2* has 3 human homologs: *FADS1*, *FADS2*, and *FADS3*. *FADS1* and *FADS2* are both associated with IBD but the gene family was only counted once (Supplemental Table S11).

Mouse RNA-seq reads were aligned to the mouse genome (mm9) using TopHat v2.1.0 using *de novo* splice junction mapping with default settings. Normalized fragments per kilobase of transcript per million mapped reads (FPKM) expression values were obtained for genes via Cufflinks, and pairwise differential gene expression tests were carried out with Cuffdiff v2.2.1.3 (Trapnell et al. 2012) using multi-read correct, bias correction and read group datasets. The default significance threshold of FDR < 5% was used for each comparison. To assess the association of differential DHS, H3K4me1 and H3K27ac and nearby gene expression differences in the presence and absence of a microbiota, we linked chromatin marks found within 10kb upstream or downstream of the nearest expressed gene (as defined by RNA-seq; minimum alignment count of 100 reads was used to determine detectable expression) transcription start site with that gene using GREAT (McLean et al. 2010) version 3.0.0 (Single nearest gene definition) for putative regulation. The distributions of fold-change FPKM values in the presence and absence of a microbiota were compared to the distributions of all 9,173 expressed genes by a two-sided Kolmogorov-Smirnov test. GO enrichments were performed using DAVID 6.7 (Huang da et al. 2009a; Huang da et al. 2009b). IBD and NEC gene

expression summary tables were derived from published studies (Arijs et al. 2009; Haberman et al. 2014; Tremblay et al. 2016).

ChIP-seq and DNase-seq Bioinformatics:

Mouse ChIP-seq reads were aligned to the mouse genome (mm9) using Bowtie2 v2.2.6 with default settings. ChIP peaks were called using the appropriate aligned input reads as the control file with MACS2 callpeak Galaxy version 2.1.0.20151222.0 with FDR <5% as the peak detection threshold. Sequencing depth normalization was performed in two ways: DESeq was used for sequencing depth normalization, variance fitting, and pairwise differential analysis (Anders and Huber 2010) and bamCoverage (Galaxy version 2.2.3.0) using RPKM normalization.

To identify differential H3K27ac and H3K4me1 sites, H3K27ac and H3K4me1 ChIP-seq peak calls were merged using the same parameters as in DNase-seq analysis except using a FDR < 1%. Raw counts were pulled from BAM files and used for calling differential peaks using R packaged DESeq2 v1.10.1 (Love et al. 2014). Motif enrichment in promoters near differential H3K27ac regions were generated using the single nearest gene definition in GREAT (McLean et al. 2010) v3.0.0 and limiting the regulatory domain to 10kb. Heat maps and average signal graphs were generated by aligning the average signals around the median number of MACS2 called peaks (See Supplemental Table S5): Supplemental Fig. S4G,H (CV DHS rep 2 peaks), Fig. 4A,B (GF HNF4A-ChIP rep 3 peaks), Supplemental Fig. S6A (CV HNF4A-ChIP rep 3 peaks), Supplemental Fig. S6C (CV HNF4A-ChIP rep 3 peaks minus GF HNF4A-ChIP rep 3 peaks). Peaks generated from sequencing noise were omitted from these analyses by MACS2 or manually. Gene associations for GF and CV HNF4A peaks were generated using the replicate with the largest number of MACS2 called peaks using the single nearest gene definition in GREAT (McLean et al. 2010) v3.0.0 and limiting the regulatory domain to 10kb. Repeat masked FASTA sequences extracted from the noted regions were submitted for *de*

novo transcription factor binding site analysis using MEME-suite (Bailey et al. 2009) (Fig. 3E,F). Specific transcription factor binding site analysis (Fig. 4D) was performed using HOMER(Heinz et al. 2010). The hierarchal clustering and heat map representation of PWM enrichment was generated with Cluster v3.0. Deming linear regression analysis was used to determine significant increases in H3K27ac, H3K4me1, and DNase-seq signal around HNF4A binding sites. Pairwise comparison of ChIP-seq signal (Supplemental Fig. S5) from individual replicates was performed by generating read counts as determined by HTSeq Galaxy Version 0.6.1.galaxy1 (Anders et al. 2015) using intersection (nonempty) and non-stranded parameters with a minimum alignment quality of 10. Reads were counted at MACS2 peaks from the GF3 replicate (GFrep3) and GF4 (GFrep4) replicate for HNF4A and HNF4G respectively.

Mouse DNase-seq reads were aligned using Bowtie version 0.12.0, with 2 mismatches allowed and mapping up to 4 sites. The output BAM files were transformed to bed files. Blacklist regions and PCR artifacts were then filtered from bed files. DNase hypersensitivity sites narrow peak calls were generated from MACS2 (version 2.1.0.20140616, https://github.com/taoliu/MACS/), with FDR <1%. To identify differential DHS sites, DHS peak calls for each condition were merged and windowed as described in ChIP-seq analysis. Raw sum counts for each base-pair's DNase-seq signals within each 300 bp window from each replicates were input into R package DESeq2 v1.10.1(Love et al. 2014) and differential peaks were identified using FDR <5% (no differential peak calls found) and $p < 1\%$.

SUPPLEMENTAL FIGURES

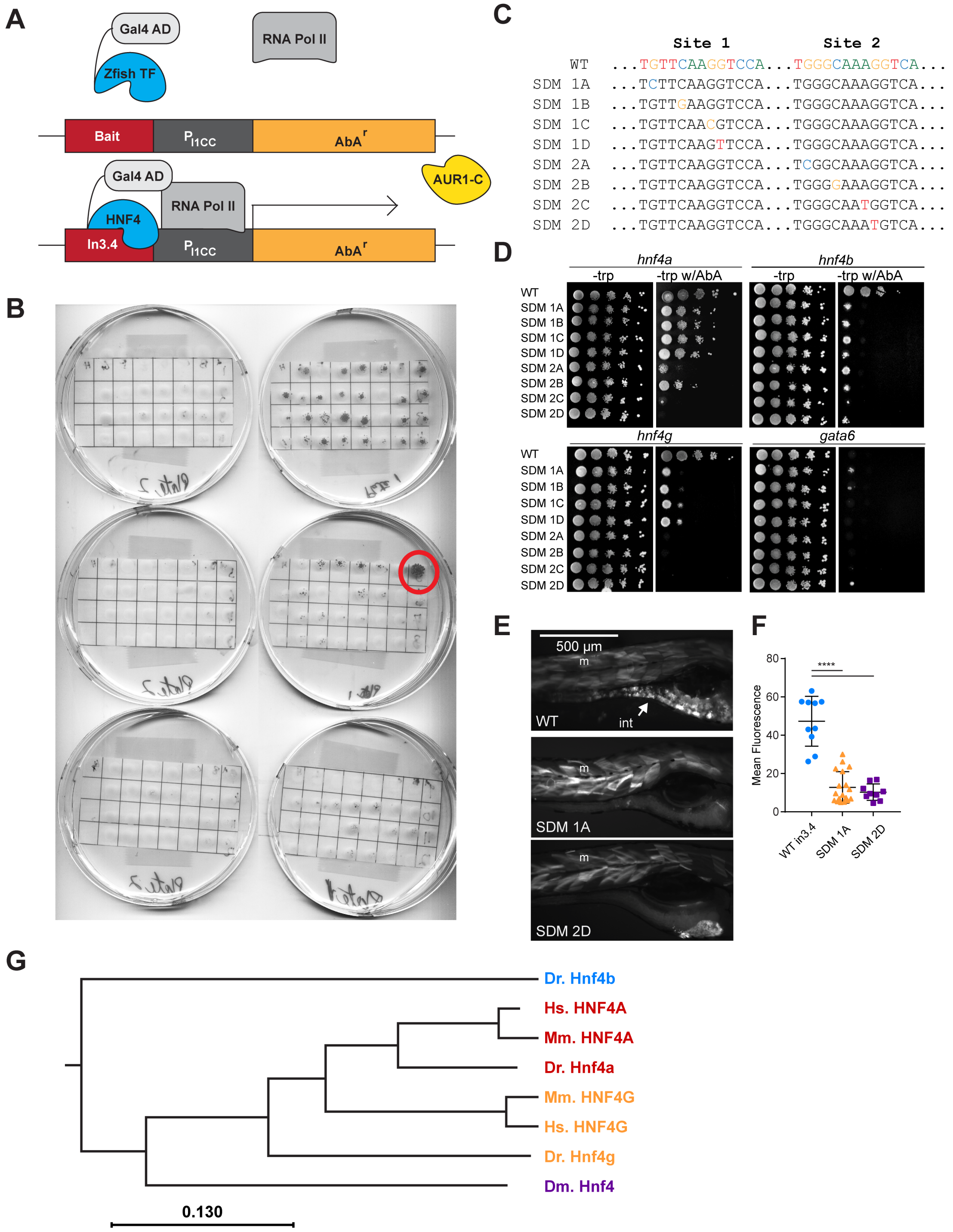

FIGURE S1:

The *hnf4* family of transcription factors bind specifically to a microbiota suppressed zebrafish enhancer. (A) Schematic showing the molecular mechanism of the yeast 1-hybrid

(Y1H) assay. A library of 148 zebrafish transcription factors was transformed into a yeast strain that contained a reporter construct that contained the zebrafish CRR in3.4. If one of the zebrafish TFs bound to in3.4, the reporter gene will be transcribed and the yeast will permit growth on media containing the antibiotic Aureobasidin A (Aba). (B) Scanned plates from the primary Y1H screen that identified *hnf4b* (red circle) as the only transcription factor that robustly permitted yeast growth on the selective media. *hnf4a* and *hnf4g* were not included in the initial 148 TF library. After cloning these TFs into the prey vectors, we found that all three zebrafish Hnf4 family members bound to the in3.4 bait sequence and drove expression of the reporter gene (Fig. 1B). Gata6 is used as a control since it is predicted to bind in3.4 (Camp et al., 2012), but does not rescue yeast growth. However, this TF family failed to rescue yeast when in3.4 was replaced with a canonical p53 binding site control bait sequence, suggesting a sequence specific interaction (data not shown). (C) Single nucleotide site-directed mutations (SDM) within Site 1 and Site 2. Using mammalian position weight matrices (PWMs), we found HNF4 is predicted to bind at two sites within regions of in3.4 previously shown to be essential for intestinal activity in transgenic reporter assays (Fig. 1A; Camp et al. 2012). To test if these putative binding sites are the location of Hnf4 binding and essential for reporter activity, we performed site-directed mutagenesis on individual nucleotides within the two predicted Hnf4 binding sites (Fig. 1C). The nucleotides selected for SDM were predicted to impact Hnf4 binding based on previously defined PWMs for mammalian HNF4A (Fang et al. 2012). The nucleotide mutation is highlighted in a color for each SDM. (D) Images of plates from serial dilutions of a Y1H assay using WT and mutated in3.4 as bait and zebrafish *hnf4* genes and *gata6* as prey. Yeast were grown on media without the selective antibiotic to demonstrate equivalent CFUs were plated and on media containing AbA which inhibits yeast growth in the absence of an actively transcribed reporter gene. Mutations in the first predicted binding site (Site 1) resulted in severe growth attenuation of yeast transformed with the *hnf4g* and *hnf4b* prey vectors. However, yeast transformed with the *hnf4a* prey vector only had partial attenuated growth when

harboring mutations in the Site 1 sequence. Strikingly, mutations in the second predicted binding site (Site 2) resulted in failed growth of yeast transformed with all three *hnf4* prey vectors with the notable exception of Site 2.2, which only partially attenuated growth of yeast transformed with the *hnf4a* prey vector. (E) To test if the putative HNF4A site was essential for in3.4 enhancer activity, we generated new versions of the *in3.4:cfos:gfp* reporter which contained single nucleotide mutations in Site 1 or 2. These reporter constructs were injected into wild-type zebrafish to generate mosaic transgenics. Single nucleotide mutations in Site 1 or 2 of the *in3.4:cfos:gfp* reporter were sufficient to ablate in3.4 intestinal activity in zebrafish. Data shown in panels D and E establish that one or more Hnf4 family members bind in3.4 in a sequence dependent manner and that mutation of the predicted Hnf4 binding sites of this microbiota-suppressed CRR result in suppressed enhancer activity in the intestinal epithelium. (F) Chart showing the GFP fluorescence (mean ± sem) in 6dpf mosaic zebrafish injected with transposase and WT (n=10) and SDM *in3.4:cfos:gfp* Tol2 vectors (n=9 and n=17). Mean fluorescence was measured within the intestine of mosaic animals using a constant region of interest (ROI) (Kruskal-Wallis, Kruskal Wallis statistic = 20.26 and **** $p < 0.0001$). (G) HNF4 protein phylogram showing the evolutionary relationship of the HNF4s across species. Branch lengths are proportional to the amount of inferred evolutionary change. See Supplemental Table S10 for sequences used to generate the phylogram. Dm. – *Drosophila melanogaster*, Dr. – *Danio rerio*, Mm. – *Mus musculus*, Hs. – *Homo sapiens*.

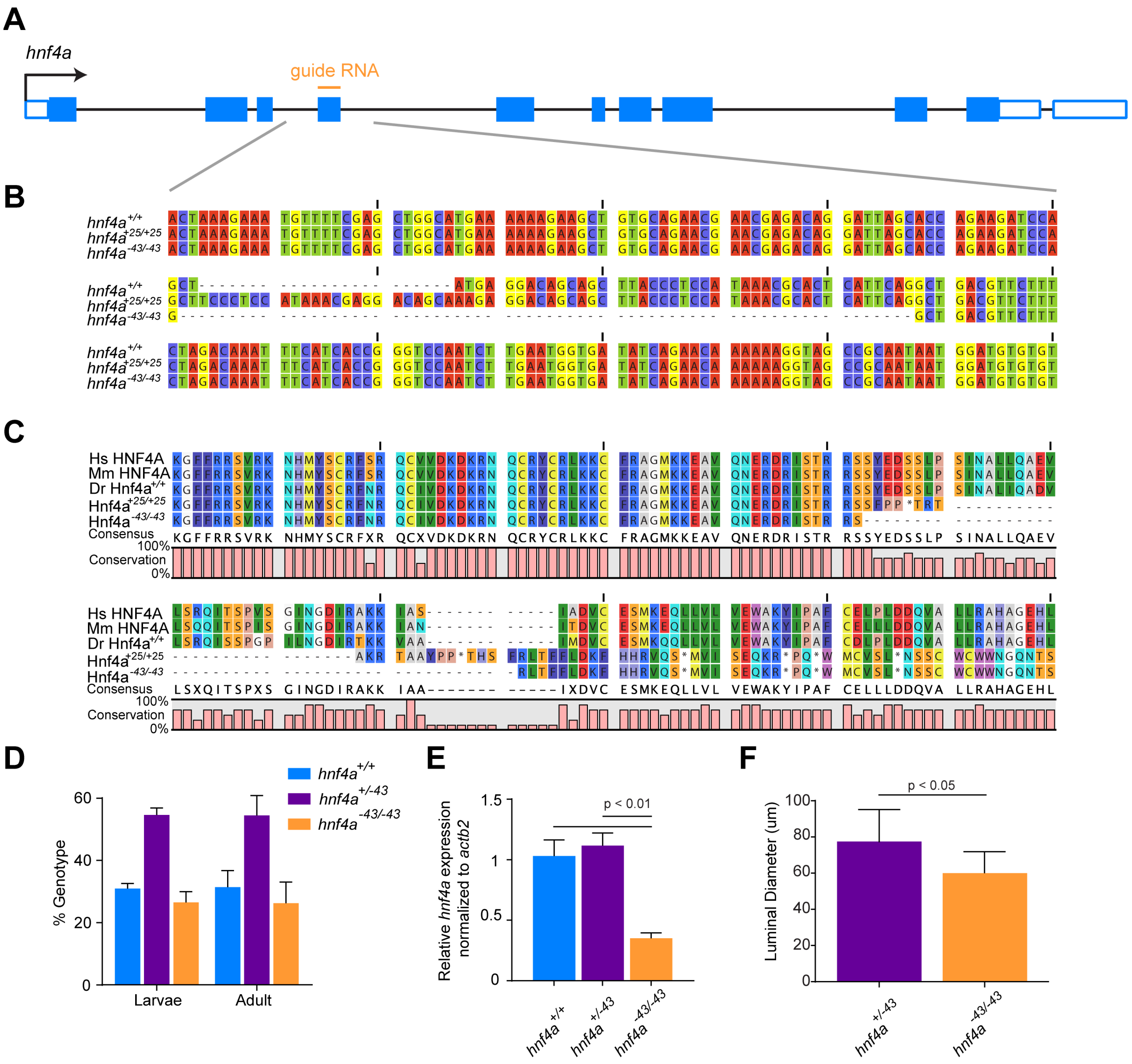

FIGURE S2:

***hnf4a*$^{-43/-43}$ mutants survive to adulthood and have reduced *hnf4a* transcript and reduced intestinal lumen size**. (A) Schematic showing the zebrafish *hnf4a* gene locus (splice form hnf4a-201) and the region that was targeted by the guide RNAs. Exons are highlighted in solid blue blocks, untranslated regions are indicated by white blocks with blue outlines, and the CRISPR targeted region is indicated by the orange line. (B) DNA sequence showing the genomic region that is mutated in the *hnf4a*$^{+25}$ and *hnf4a*$^{-43}$ allele. (C) Amino acid sequence of human, mouse, and WT and mutant zebrafish Hnf4a proteins showing sequence conservation

in the DNA binding domain and hinge domain. The *hnf4a*$^{+25/+25}$ and *hnf4a*$^{-43/-43}$ mutations are predicted to result in truncated proteins in these highly conserved domains. (D) Bar graph showing genotypes at the expected Mendelian ratios of progeny from an *hnf4a*$^{+/-43}$ heterozygous incross at both 6dpf and adult stages (mean ± sem). (E) Bar graph showing the *hnf4a* relative mRNA expression (mean ± sem) from whole *hnf4a*$^{+/+}$ (n = 4), *hnf4a*$^{+/-43}$ (n = 4) and *hnf4a*$^{-43/-43}$ (n = 4) 6dpf larvae (Two-tailed t-test, t = 4.79, 6.734, respectively and df = 6). (F) Bar graph showing the diameter of the intestinal lumen (mean ± sem) along the first segment of *hnf4a*$^{+/+}$ (n = 9) and *hnf4a*$^{-43/-43}$ (n = 8) 6dpf larvae (Two-tailed t-test, t = 2.56, df = 15 and p = 0.0219).

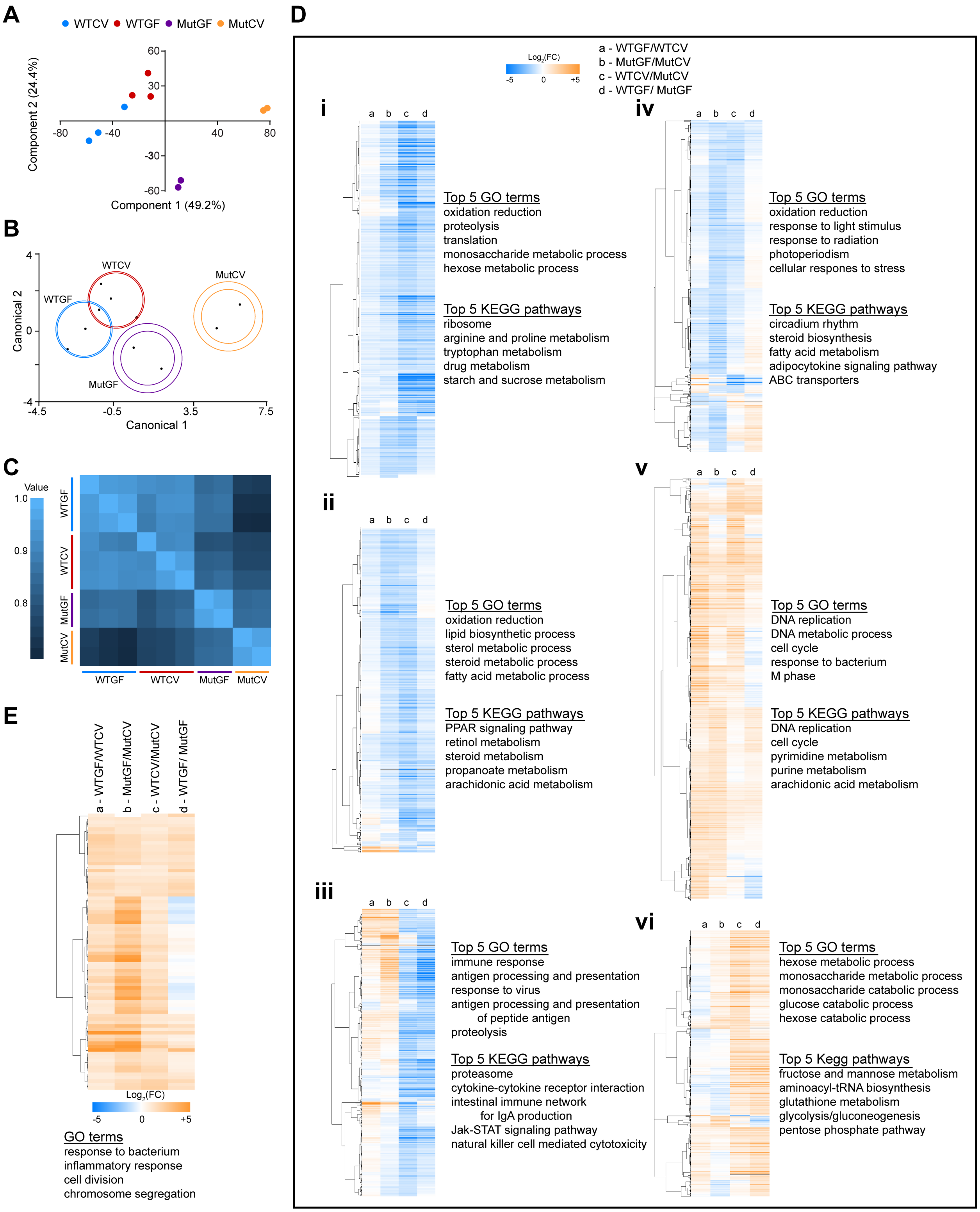
A
WTCV
WTGF
MutGF
MutCV
Component 2 (24.4%)
Component 1 (49.2%)
B
Canonical 2
Canonical 1
WTCV
WTGF
MutGF
MutCV
C
Value
WTGF
WTCV
MutGF
MutCV
D
a - WTGF/WTCV
b - MutGF/MutCV
c - WTCV/MutCV
d - WTGF/ MutGF
i
Top 5 GO terms
oxidation reduction
proteolysis
translation
monosaccharide metabolic process
hexose metabolic process
Top 5 KEGG pathways
ribosome
arginine and proline metabolism
tryptophan metabolism
drug metabolism
starch and sucrose metabolism
ii
Top 5 GO terms
oxidation reduction
lipid biosynthetic process
sterol metabolic process
steroid metabolic process
fatty acid metabolic process
Top 5 KEGG pathways
PPAR signaling pathway
retinol metabolism
steroid metabolism
propanoate metabolism
arachidonic acid metabolism
iii
Top 5 GO terms
immune response
antigen processing and presentation
response to virus
antigen processing and presentation
of peptide antigen
proteolysis
Top 5 KEGG pathways
proteasome
cytokine-cytokine receptor interaction
intestinal immune network
for IgA production
Jak-STAT signaling pathway
natural killer cell mediated cytotoxicity
iv
Top 5 GO terms
oxidation reduction
response to light stimulus
response to radiation
photoperiodism
cellular respones to stress
Top 5 KEGG pathways
circadium rhythm
steroid biosynthesis
fatty acid metabolism
adipocytokine signaling pathway
ABC transporters
v
Top 5 GO terms
DNA replication
DNA metabolic process
cell cycle
response to bacterium
M phase
Top 5 KEGG pathways
DNA replication
cell cycle
pyrimidine metabolism
purine metabolism
arachidonic acid metabolism
vi
Top 5 GO terms
hexose metabolic process
monosaccharide metabolic process
monosaccharide catabolic process
glucose catabolic process
hexose catabolic process
Top 5 Kegg pathways
fructose and mannose metabolism
aminoacyl-tRNA biosynthesis
glutathione metabolism
glycolysis/gluconeogenesis
pentose phosphate pathway
E
a - WTGF/WTCV
b - MutGF/MutCV
c - WTCV/MutCV
d - WTGF/ MutGF
GO terms
response to bacterium
inflammatory response
cell division
chromosome segregation

FIGURE S3:

Hnf4a maintains transcriptional homeostasis in the presence of a microbiota in zebrafish digestive tracts. (A) Principle components analysis (PCA) using a wide estimation method showing the relative similarities of mRNA-seq transcript abundances in digestive tracts from WT germ-free (WTGF, blue), WT conventionalized (WTCV, purple), *hnf4a*$^{-/-}$ germ-free (MutGF, red), and *hnf4a*$^{-/-}$ conventionalized (MutCV, orange) 6dpf zebrafish. (B) Discriminant analysis using wide linear parameters plot showing the relative similarities and statistical groupings of mRNA-seq transcript abundances in digestive tracts from WTGF (blue), WTCV (purple), MutGF (red), and MutCV (orange) 6dpf zebrafish. The inner ellipse of each group signifies the 95% confidence interval to contain true mean of that group and the outer ellipse signifies 50% of the population within the group is contained within ellipse. (C) Heat map showing the relative similarities of mRNA-seq transcript abundances in digestive tracts from WTGF (blue), WTCV (purple), MutGF (red), and MutCV (orange) 6dpf zebrafish. (D) Heat maps referred to in Fig. 3B showing the $\log_2$ (FC) relative expression of sublists of differentially regulated genes. The top 5 enriched GO terms and top 5 enriched KEGG pathways for each sublist of genes is included to the left of each heat map. The 4 columns are: WTCV/WTGF, MutCV/MutGF, MutCV/WTCV, and MutGF/WTCV, respectively. (E) Heat map showing the Log_2 (FC) relative expression of 86 genes that have an exacerbated microbiota induction in the *hnf4a*$^{-/-}$ digestive tracts. The GO term enrichment for this set of genes is included to the left of the heat map.

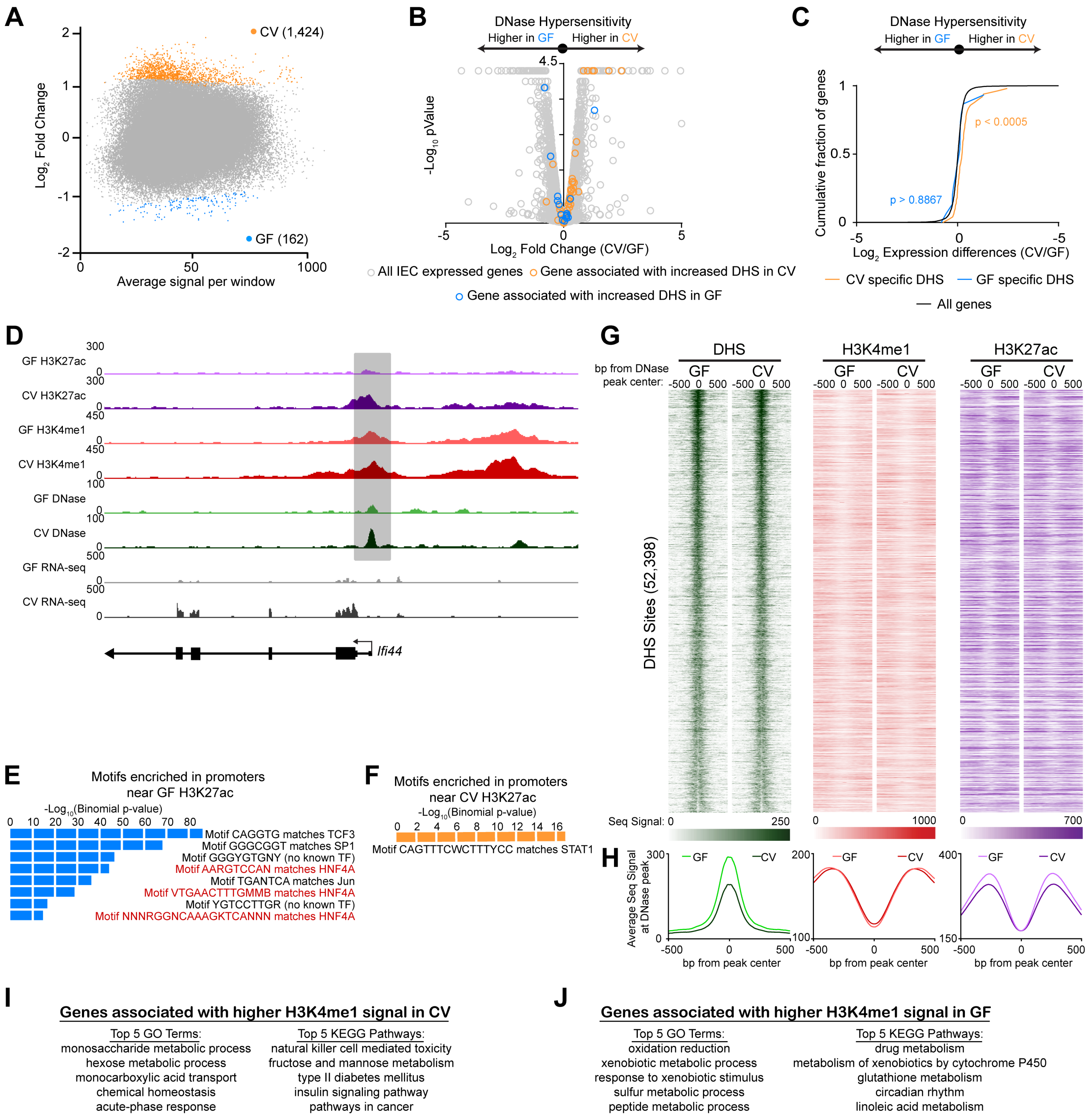

FIGURE S4:

HNF4A and STAT1 binding sites are enriched with within promoters of microbiota suppressed and induced genes, respectively. (A) MA plots from DESeq2 analysis of DNase hypersensitivity from GF and CV mouse jejunal IECs. Colored dots signify regions enriched for a histone mark in GF (blue) or CV (orange) jejunal IECs. We detect 162 unique DHSs in GF and

1,424 unique DHS in CV (Supplemental Table S6). These results were generated using a less stringent significance test ($p < 0.01$) rather than FDR < 0.01 utilized in our other comparisons in this study, therefore, we are less confident in these results. Using a FDR cutoff of < 0.01, none of these regions are significantly different. (B) Volcano plots showing pairwise comparison of RNA expression between GF and CV jejunal IECs. Blue and orange dots represent genes associated with a region enriched for DNase signal in GF or CV, respectively. (C) Two-sided Kolmogorov-Smirnov goodness-of-fit test shows a positive relationship between the presence of a DHS in a specific colonization state and increased transcript abundance in that same colonization state. The y-axis shows the cumulative fraction of genes linked to microbiota induced DHSs. Deviation from the null expectation that linked genes display a normal distribution on a fold change of 1 (black line) suggests that microbiota induced DHSs are enriched near genes of higher expression upon microbiota colonization. Though the Kolmogrov-Smirnov test may not be sufficient for such a small number of sites, these results suggest that IEC gene expression responses to microbiota are not explained by changes in chromatin accessibility. (D) Representative signal track highlighting a microbiota induced gene associated with a CV-specific DHS and regions enriched for H3K4me1 and H3K27ac CV (*Ifi44*, *Interferon induced protein 44).* Bar graphs showing the enrichment for specific TF bindings sites within promoters for genes associated with increased H3K27ac regions in GF (E) or CV (F). (G) Heat maps of the replicate average DNase signal, H3K27ac signal, and H3K4me1 signal at individual CV DHS sites. Despite the MA plots indicating an increase in DNase-seq signal in CV conditions (Fig.3A and Supplemental Fig. S4A), we find these heat maps do not show this trend. (H) Line plots showing the average GF (light-colored line) and CV (dark-colored line) -seq signal for the indicated TF, histone mark or DHS at the 1000 bp flanking DHS sites found in CV. The average DNase-signal at all DHS sites is significantly increased in GF compared CV. Similarly, the average H3K27ac signal at these DHS sites shows a significant increase in H3K27ac signal in GF compared to CV by the Whitney-Mann U test. We do not see this trend in H3K4me1. The

tentative discrepancies in results between the MA plots and average signal plots can be explained by inherent differences in DESeq2 and average signal analysis. DESeq2 performs a powerful statistical test to determine differential signal and therefore is a more ideal type of analysis to identify enrichment of DHS or histone marks based on variation and average signal of replicates within a set window. We performed the second type of analysis which shows the average signal at a given base pair relative to the center of a ChIP peak because DESeq2 would be an inappropriate analysis for downstream applications in which the GF conditions had an overwhelming signal compared to CV (i.e., HNF4A and HNF4G ChIP-seq, Fig. 4). Instead, we find this second type of analysis is only useful when comparing the signals or relative signals from the same condition at two different sets of genomic locations (see Supplemental Fig. 5F,G). Furthermore, the average number and median number of DHS sites, H3K27ac peaks and H3K4me1 peaks as determined by MACS2 were similar between GF and CV conditions; this was not true for HNF4A or HNF4G peaks (Supplemental Table S5). Based on our stringent DESeq2 analysis (FDR < 0.01) and because of the similar number of MACS2 peaks, we do not believe the average H3K27ac and DHS signal differences at DHS sites between CV and GF conditions is biologically or technically relevant to our conclusions. (I-J) GO terms and KEGG pathways enriched in genes associated with differential H3K4me1 sites shown in Fig. 3I.

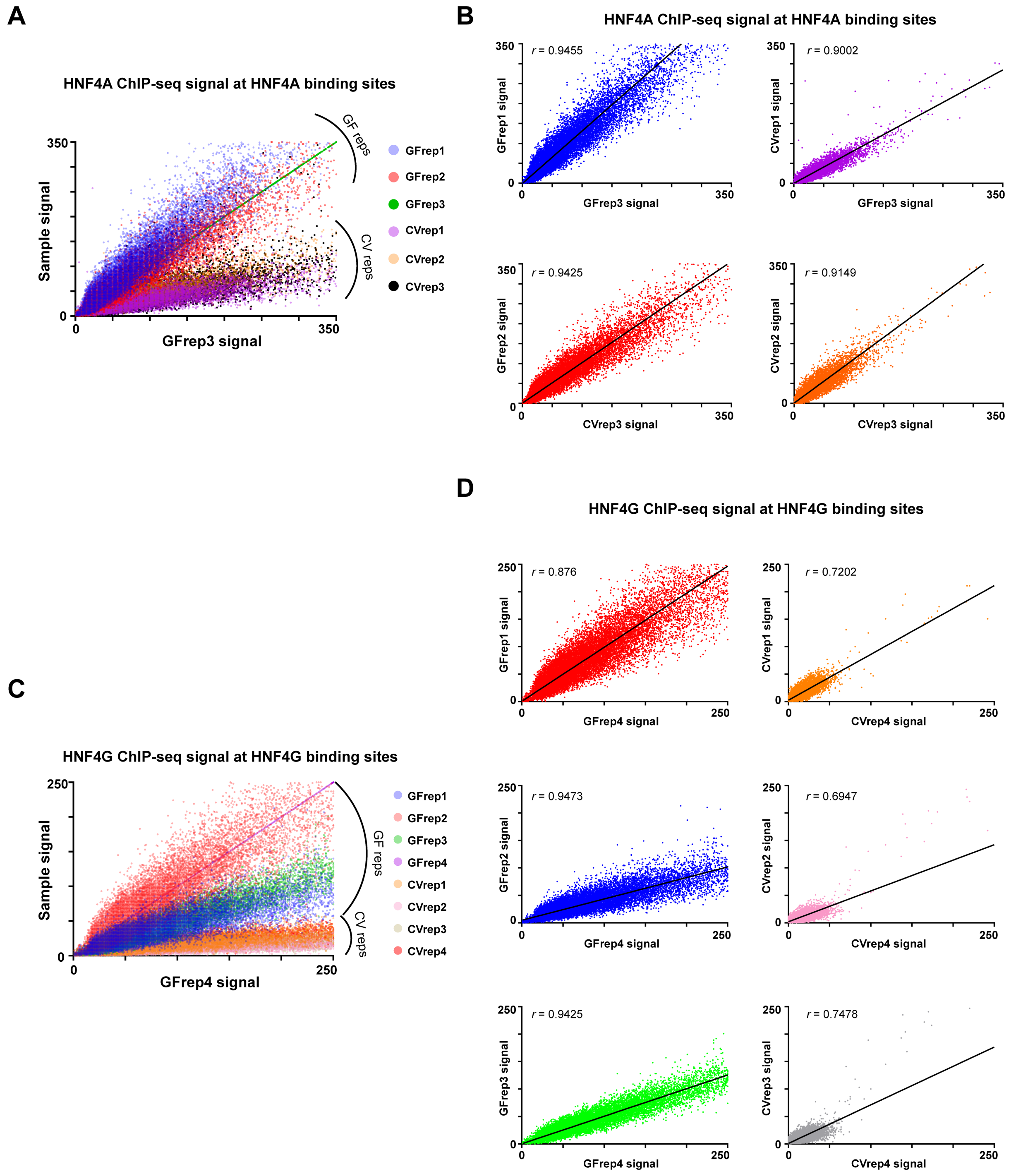

FIGURE S5:

HNF4 GF ChIP-Seq replicates have reproducibly higher signal than HNF4 CV Chip-seq replicates. (A) Grouped pairwise comparison of all HNF4A ChIP-seq signal at HNF4A binding sites compared to GFrep3 (third HNF4A GF replicate). (B) Individual pairwise comparison of

HNF4A ChIP-seq signal at binding sites compared to GFrep3 (third HNF4A GF replicate) or CVrep3 (third HNF4A CV replicate). (C) Grouped pairwise comparison of all HNF4G ChIP-seq signal at HNF4G binding sites compared to GFrep4 (fourth HNF4G GF replicate). (D) Individual pairwise comparison of HNF4G ChIP-seq signal at HNF4G binding sites compared to GFrep4 (fourth HNF4G GF replicate) or CVrep4 (fourth HNF4G CV replicate). The correlation coefficient (*r*) is provided for each graph. We believe the HNF4G CV *r* value was substantially lower than the other reported HNF4A and HNF4G correlation coefficients because of the very low signal-to-noise ratio among these replicates.

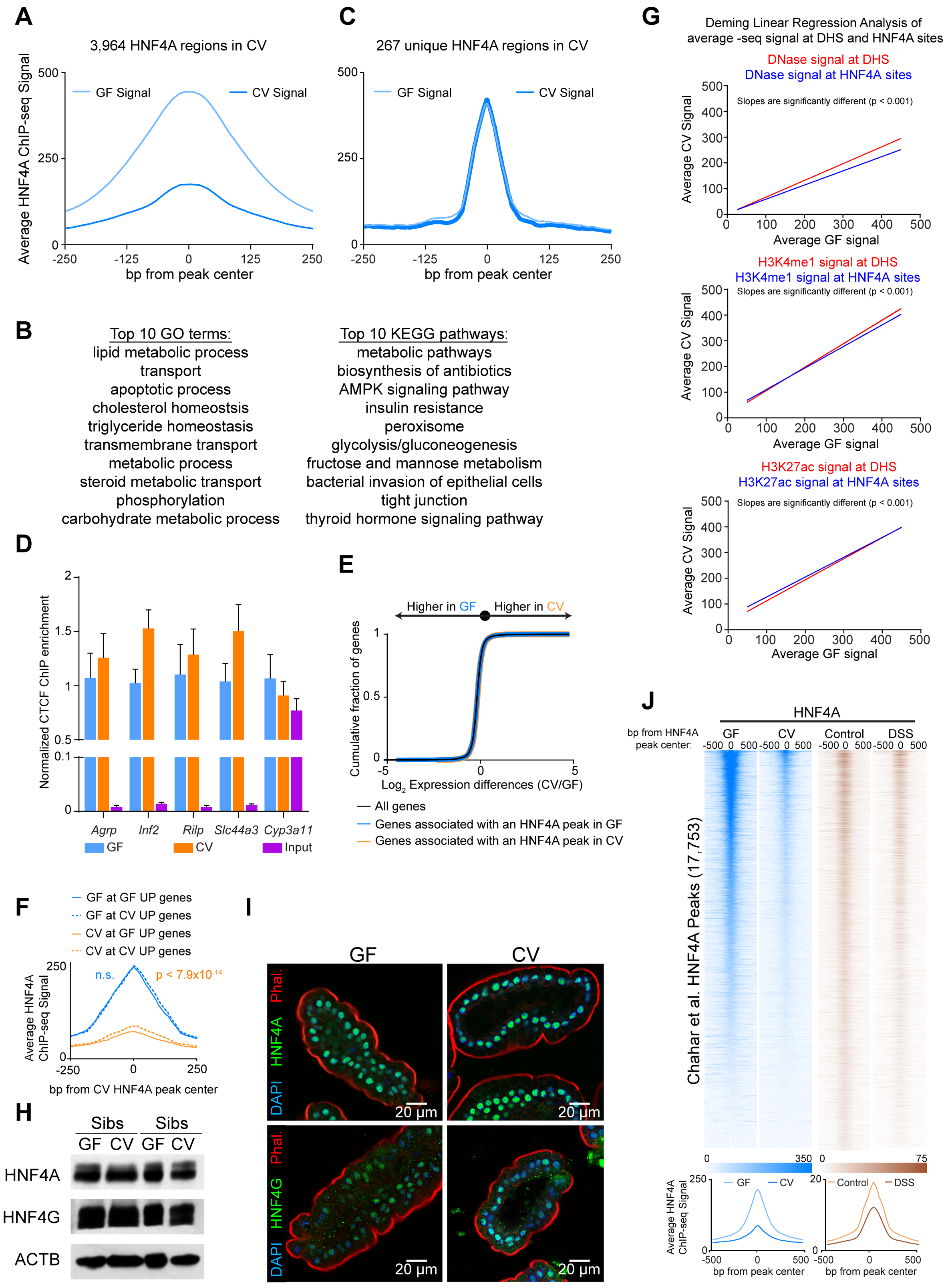
A
3,964 HNF4A regions in CV
GF Signal
CV Signal
Average HNF4A ChIP-seq Signal
bp from peak center
C
267 unique HNF4A regions in CV
GF Signal
CV Signal
bp from peak center
G
Deming Linear Regression Analysis of
average -seq signal at DHS and HNF4A sites
DNase signal at DHS
DNase signal at HNF4A sites
Slopes are significantly different (p < 0.001)
Average CV Signal
Average GF signal
H3K4me1 signal at DHS
H3K4me1 signal at HNF4A sites
Slopes are significantly different (p < 0.001)
H3K27ac signal at DHS
H3K27ac signal at HNF4A sites
Slopes are significantly different (p < 0.001)
B
Top 10 GO terms:
lipid metabolic process
transport
apoptotic process
cholesterol homeostsis
triglyceride homeostasis
transmembrane transport
metabolic process
steroid metabolic transport
phosphorylation
carbohydrate metabolic process
Top 10 KEGG pathways:
metabolic pathways
biosynthesis of antibiotics
AMPK signaling pathway
insulin resistance
peroxisome
glycolysis/gluconeogenesis
fructose and mannose metabolism
bacterial invasion of epithelial cells
tight junction
thyroid hormone signaling pathway
D
Normalized CTCF ChIP enrichment
Agrp
Inf2
Rilp
Slc44a3
Cyp3a11
GF
CV
Input
E
Higher in GF
Higher in CV
Cumulative fraction of genes
Log2 Expression differences (CV/GF)
All genes
Genes associated with an HNF4A peak in GF
Genes associated with an HNF4A peak in CV
J
HNF4A
bp from HNF4A peak center:
GF
CV
Control
DSS
Chahar et al. HNF4A Peaks (17,753)
Average HNF4A ChIP-seq Signal
bp from peak center
F
GF at GF UP genes
GF at CV UP genes
CV at GF UP genes
CV at CV UP genes
n.s.
p < 7.9x10-14
Average HNF4A ChIP-seq Signal
bp from CV HNF4A peak center
I
GF
CV
Phal.
HNF4A
DAPI
HNF4G
20 μm
H
Sibs
GF CV GF CV
HNF4A
HNF4G
ACTB

FIGURE S6:

Microbiota suppress HNF4A and HNF4G activity without overtly impacting protein levels or localization. (A) Line plots showing the average GF HNF4A (light-colored line) and CV HNF4A (dark-colored line) -seq signal at the 500 bp flanking HNF4A sites found in CV. We chose to forgo DESeq2 analysis with the HNF4 ChIP samples because of the obvious signal bias toward GF conditions would skew the results and fail to provide useful statistical significances. We therefore performed all subsequent downstream analyses using average signal surrounding ChIP peaks. (B) Top 10 GO terms and top 10 KEGG pathways for genes associated with CV HNF4A sites. These enrichments indicate CV retains HNF4A binding near genes that are associated with canonical HNF4A and intestinal function. (C) Line plots showing the average GF HNF4A (light-colored line) and CV HNF4A (dark-colored line) -seq signal at the 500 bp flanking "Unique" HNF4A sites found in CV based on MACS2 peaks and peak coordinate intersections. Note that HNF4A ChIP-seq signal at these sites is similar in GF and CV. (D) Bar graph of CTCF ChIP-PCR results at different loci (n = 2 per condition). Loci were chosen based on publically available mouse intestinal CTCF tracks on the UCSC genome browser. The relative enrichment of CTCF binding at *Agrp*, *Inf2*, *Rilp*, *Slc44a3,* and *Cyp3a11* loci was normalized to CTCF signal at the *Neurog1* locus (negative control). (E) Two-sided Kolmogorov-Smirnov goodness-of-fit test shows no relationship between the presence of an HNF4A site in GF (blue) or CV (orange) and increased transcript abundance in that same colonization state. Our zebrafish RNA-seq data predict that HNF4A directly or indirectly regulates both microbiota suppressed and induced genes. In accord, we did not find an overt association with HNF4A binding sites and microbiota suppressed or induced genes. (F) Line plots showing the average signal of GF (blue) and CV (orange) HNF4A ChIP-seq RPKM-normalized signal at the 500 bp flanking HNF4A peaks associated with microbiota-suppressed genes (solid) and microbiota-induced genes

(dotted). Statistical measurements were performed using a two-tailed Mann-Whitney test. (G) Deming linear regression of the average GF and CV ChIP/DHS signals at HNF4A sites and DHS sites. To determine if histone marks correlate with the loss of HNF4A signal in CV conditions, we aligned the average histone ChIP-seq signals and DHS signals to the 28,901 GF HNF4A sites. As expected, we found both GF and CV H3K27ac and H3K4me1 signals were enriched on the flanks of the HNF4A peaks while DHS signal was enriched near the center of the HNF4A peaks (Fig. 4A,B). Interestingly, we observed that colonization resulted in a reduction in H3K4me1 signal at HNF4A sites, a trend we did not see when comparing signals at all DHS sites (Fig.4B and Supplemental Fig. S4H). Active CV enhancer signals and CV DHS signals were also reduced at HNF4A sites (Fig.4B). However, the average signals of these genomic marks were already reduced upon colonization at DHS sites (Supplemental Fig. S4H). Therefore, to determine if the presence of an HNF4A site corresponded to a reduction in enhancer activity or chromatin accessibility upon microbiota colonization, we performed Deming linear regression. If the slopes of the Deming linear regression are significantly different, we can conclude that the relative signal at the two different sets of genomic locations is significantly different. We found that HNF4A sites correspond with increased H3K27ac, H3K4me1 and DHS signal in GF compared to these same chromatin marks in CV. (H) Western blots of HNF4A and HNF4G from GF and CV mouse jejunal IECs. Beta-actin was used as a loading control. (I) Representative confocal immunofluorescence (n = 2 per condition) optical section of wholemount GF and CV mouse jejunal villi stained for phalloidin (red), HNF4A/HNF4G (green) and DAPI (blue). (J) Heat maps and line plots of showing the average GF and CV HNF4A ChIP-signal from primary mouse jejunal IECs (blue) and the single replicates of control and DSS-treated HNF4A ChIP-signal from primary colonocytes. When we assessed the average GF and CV jejunal HNF4A signals at the colonic HNF4A peaks from this previous study, we observed jejunal HNF4A signal at the majority of colonic peaks and reduced CV HNF4A averaged signal

compared to GF. This finding reveals that the HNF4A cistrome in the small and large intestine is remarkably similar, and that HNF4A occupancy at many of these sites is similarly reduced by microbiota and inflammation.

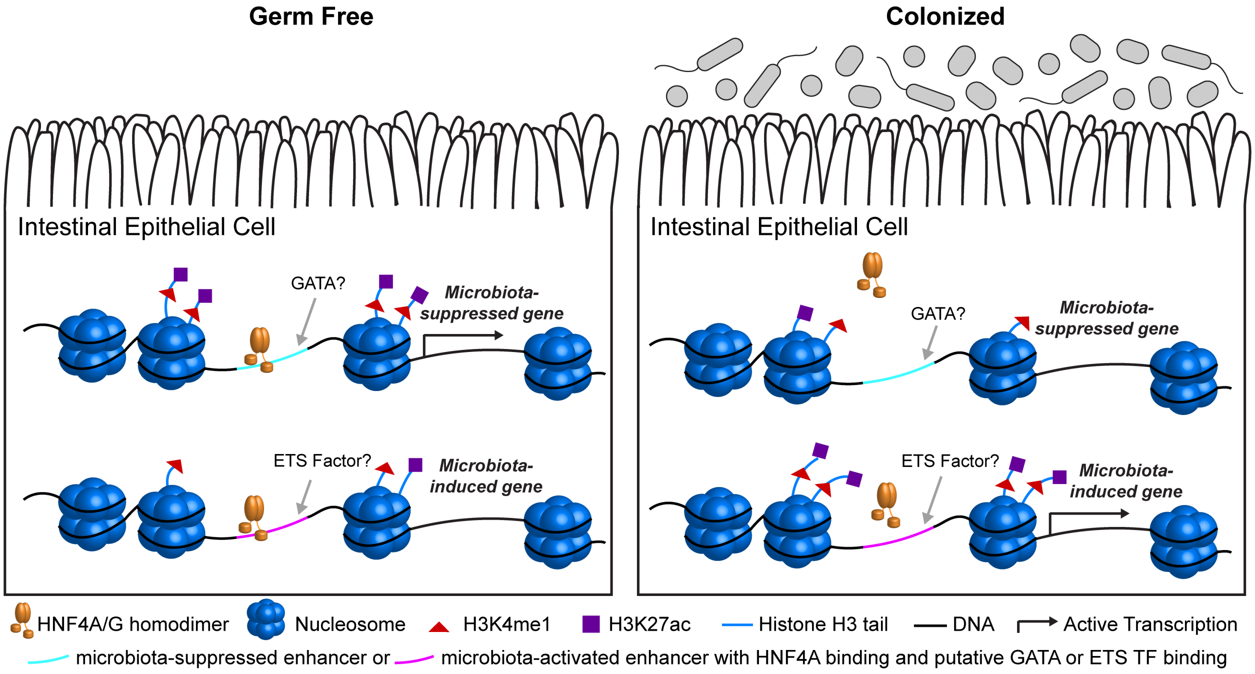

FIGURE S7:

Model of microbiota regulation of host gene transcription through modification of enhancer activity and suppression of HNF4A DNA binding. A model depicting the key findings from the manuscript. Microbiota-suppressed H3K27ac and H3K4me1 marks (microbiota-suppressed enhancers) are significantly enriched near genes that are downregulated by the microbiota. Microbiota-induced H3K27ac and H3K4me1 marks (microbiota-induced enhancers) are significantly enriched near genes that are upregulated upon microbiota colonization. Also, following microbiota colonization, HNF4A DNA binding is reduced across the genome. This reduced occupancy occurs near genes that are both microbiota-induced and microbiota-suppressed genes. GATA factor binding sites are located near HNF4A binding sites that associate with microbiota-suppressed genes and microbiota-suppressed enhancers. ETS factor binding sites are located near HNF4A binding sites that associate with microbiota-induced genes and microbiota-induced enhancers.

SUPPLEMENTAL REFERENCES

Anders S, Pyl PT, Huber W. 2015. HTSeq--a Python framework to work with high-throughput sequencing data. *Bioinformatics* **31**: 166-169.

Arijs I, De Hertogh G, Lemaire K, Quintens R, Van Lommel L, Van Steen K, Leemans P, Cleynen I, Van Assche G, Vermeire S et al. 2009. Mucosal gene expression of antimicrobial peptides in inflammatory bowel disease before and after first infliximab treatment. *PLoS One* **4**: e7984.

Bailey TL, Boden M, Buske FA, Frith M, Grant CE, Clementi L, Ren J, Li WW, Noble WS. 2009. MEME SUITE: tools for motif discovery and searching. *Nucleic Acids Res* **37**: W202-208.

Boyle G, Richter K, Priest HD, Traver D, Mockler TC, Chang JT, Kay SA, Breton G. 2017. Comparative Analysis of Vertebrate Diurnal/Circadian Transcriptomes. *PLoS One* **12**: e0169923.

Camp JG, Frank CL, Lickwar CR, Guturu H, Rube T, Wenger AM, Chen J, Bejerano G, Crawford GE, Rawls JF. 2014. Microbiota modulate transcription in the intestinal epithelium without remodeling the accessible chromatin landscape. *Genome Res* **24**: 1504-1516.

Fang B, Mane-Padros D, Bolotin E, Jiang T, Sladek FM. 2012. Identification of a binding motif specific to HNF4 by comparative analysis of multiple nuclear receptors. *Nucleic Acids Res* **40**: 5343-5356.

Haberman Y, Tickle TL, Dexheimer PJ, Kim MO, Tang D, Karns R, Baldassano RN, Noe JD, Rosh J, Markowitz J et al. 2014. Pediatric Crohn disease patients exhibit specific ileal transcriptome and microbiome signature. *J Clin Invest* **124**: 3617-3633.

Heinz S, Benner C, Spann N, Bertolino E, Lin YC, Laslo P, Cheng JX, Murre C, Singh H, Glass CK. 2010. Simple combinations of lineage-determining transcription factors prime cis-regulatory elements required for macrophage and B cell identities. *Mol Cell* **38**: 576-589.

Hu Y, Flockhart I, Vinayagam A, Bergwitz C, Berger B, Perrimon N, Mohr SE. 2011. An integrative approach to ortholog prediction for disease-focused and other functional studies. *BMC Bioinformatics* **12**: 357.

Huang da W, Sherman BT, Lempicki RA. 2009a. Bioinformatics enrichment tools: paths toward the comprehensive functional analysis of large gene lists. *Nucleic Acids Res* **37**: 1-13.

Huang da W, Sherman BT, Lempicki RA. 2009b. Systematic and integrative analysis of large gene lists using DAVID bioinformatics resources. *Nat Protoc* **4**: 44-57.

Jao LE, Wente SR, Chen W. 2013. Efficient multiplex biallelic zebrafish genome editing using a CRISPR nuclease system. *Proc Natl Acad Sci U S A* **110**: 13904-13909.

Love MI, Huber W, Anders S. 2014. Moderated estimation of fold change and dispersion for RNA-seq data with DESeq2. *Genome Biol* **15**: 550.

McLean CY, Bristor D, Hiller M, Clarke SL, Schaar BT, Lowe CB, Wenger AM, Bejerano G. 2010. GREAT improves functional interpretation of cis-regulatory regions. *Nat Biotechnol* **28**: 495-501.

Pham LN, Kanther M, Semova I, Rawls JF. 2008. Methods for generating and colonizing gnotobiotic zebrafish. *Nat Protoc* **3**: 1862-1875.

Song L, Crawford GE. 2010. DNase-seq: a high-resolution technique for mapping active gene regulatory elements across the genome from mammalian cells. *Cold Spring Harb Protoc* **2010**: pdb prot5384.

Tremblay E, Thibault MP, Ferretti E, Babakissa C, Bertelle V, Bettolli M, Burghardt KM, Colombani JF, Grynspan D, Levy E et al. 2016. Gene expression profiling in necrotizing enterocolitis reveals pathways common to those reported in Crohn's disease. *BMC Med Genomics* **9**: 6.

Westerfield M. 2000. *The Zebrafish Book. A guide for the laboratory use of zebrafish (Danio rerio).* Univ. of Oregon Press, Eugene, OR.
