# Supplementary material for: Microbiota regulate intestinal epithelial gene expression by suppressing the transcription factor Hepatocyte nuclear factor 4 alpha
Source: Genome Res. 2017 Jul;27(7):1195–206. doi: 10.1101/gr.220111.116 (PMC5495071; doi:10.1101/gr.220111.116)
Supplement: Supplemental Material [file supp_gr.220111.116_Supplemental_Table_S5.docx]

**Supplemental Table S5: Total number of MACS2 peak calls per ChIP per replicate (related to Fig. 3 and Fig. 4).**

Number of peaks indicated are raw results generated from MACS2 and peaks generated by background signal/noise have not been filtered out. All peaks generated by background sequencing noise were removed manually for downstream analysis.

|  | **DHS** | **H3K4me1** | **H3K27ac** | **HNF4A** | **HNF4G** |
| --- | --- | --- | --- | --- | --- |
| **GF rep 1** | 89,507 | 132,275 | 82,935 | 36,850 | 12,465 |
| **GF rep 2** | 61,355 | 131,876 | 83,336 | 27,016 | 35,302 |
| **GF rep 3** |  | 137,770 |  | 29,070 | 15,934 |
| **GF rep 4** |  |  |  |  | 27,816 |
| **CV rep 1** | 70,794 | 139,559 | 83,661 | 1,889 | 101 |
| **CV rep 2** | 52,431 | 144,137 | 81,997 | 8,473 | 18 |
| **CV rep 3** | 42,433 | 146,199 |  | 4,195 | 27 |
| **CV rep 4** |  |  |  |  | 106 |
| **GF Average** | 57,485 | 133,974 | 83,136 | 30,979 | 22,879 |
| **CV Average** | 55,219 | 143,298 | 82,829 | 4,852 | 63 |
| **GF Median** | 57,485 | 132,275 | 83,136 | 29,070 | 21,875 |
| **CV Median** | 52,431 | 144,137 | 82,829 | 4,195 | 64 |
